# The microbiome–EBV axis in carcinogenesis bridges commensal influence and viral oncogenesis

**DOI:** 10.3389/fimmu.2026.1909260

**Published:** 2026-08-10

**Authors:** Xuan Tian, Kangkang Ji, Mingqian Feng

**Affiliations:** 1 College of Biomedicine and Health, Huazhong Agricultural University, Wuhan, Hubei, China; 2 College of Life Science and Technology, Huazhong Agricultural University, Wuhan, Hubei, China; 3 Department of Respiratory and Critical Care Medicine, Binhai County People’s Hospital, Binhai Clinical College, Yangzhou University Medical College, Yancheng, Jiangsu, China

**Keywords:** diagnostic biomarkers, Epstein-Barr virus (EBV), microbiome, microbiome–EBV axis, tumorigenesis

## Abstract

This review synthesizes current evidence on the emerging role of the host microbiome in Epstein–Barr virus (EBV)-associated tumorigenesis. Moving beyond the traditional virus–host paradigm, we propose the “microbiome–EBV axis” as a conceptual framework for understanding interactions among microbial communities, microbial metabolites, EBV-infected cells, and the host tissue–immune environment during carcinogenesis. We summarize four interrelated mechanisms: microbial regulation of EBV latency and lytic reactivation, microbiome-associated remodeling of the immune microenvironment, microbial contribution to genomic and epigenetic instability, and the potential translational use of microbial signatures or microbiome-targeted interventions. Importantly, we distinguish mechanistic evidence derived from *in vitro* and animal studies, observational associations from clinical cohorts, and findings supported by clinical validation. Overall, this framework refines our understanding of EBV-associated cancers while highlighting microbiome-derived biomarkers and therapeutic strategies as promising, but still incompletely validated, areas for future investigation.

## 1 EBV–microbiome interactions in oncogenesis

Epstein-Barr virus (EBV), a near-ubiquitous herpesvirus, has long been established as an oncovirus (1). However, research has largely focused on direct virus–host interactions, with the human microbiome often treated as a background factor. This perspective does not fully explain two persistent clinical observations. Given that >95% of the global adult population harbors lifelong latent EBV, why does malignancy develop in only a small minority of infected individuals? Furthermore, why do specific EBV-associated malignancies, such as nasopharyngeal carcinoma, show marked geographical clustering rather than a random distribution?

**Figure 1:**
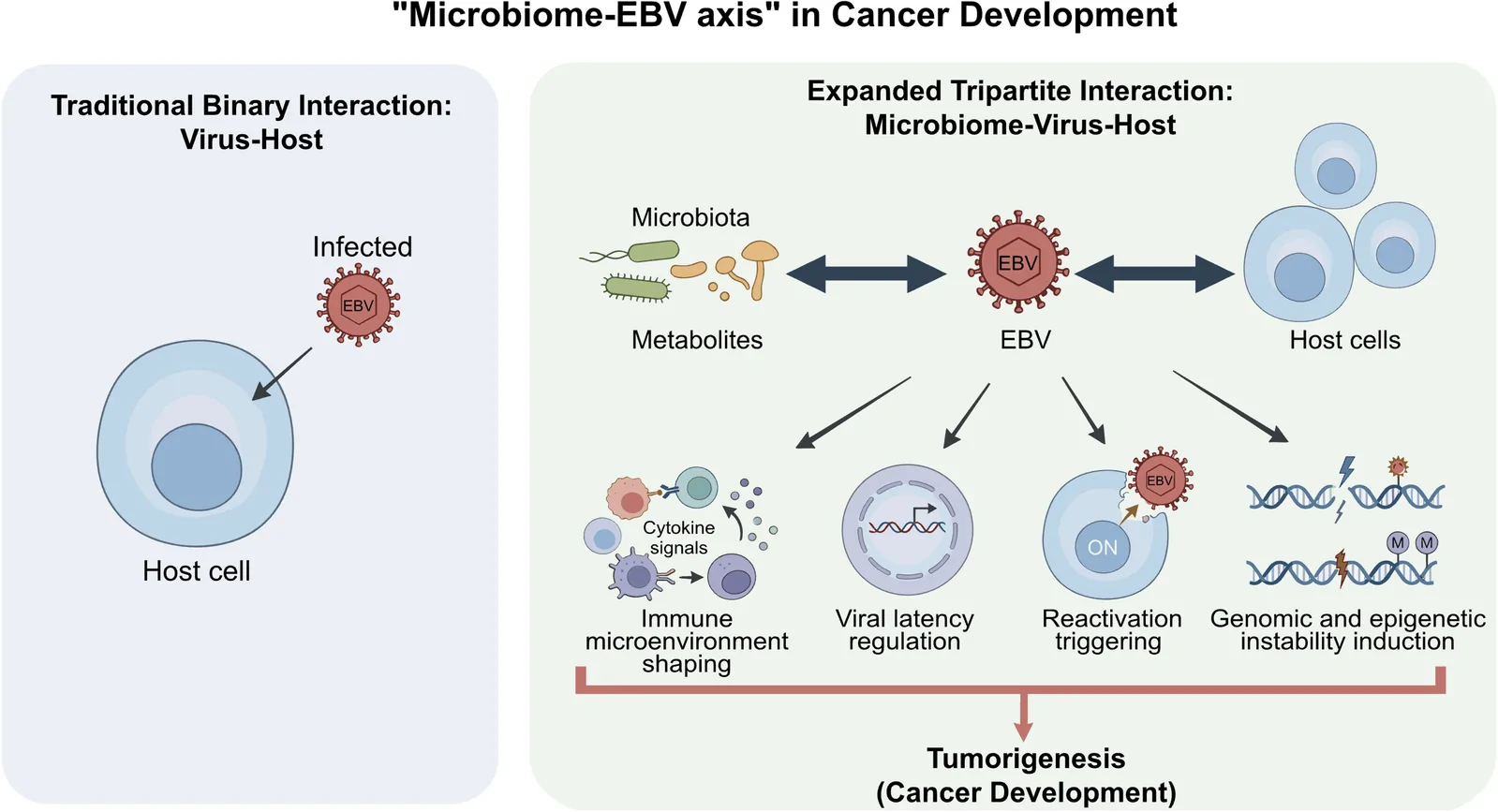
Conceptual schematic of the “Microbiome–EBV axis” in tumorigenesis. The left side shows the traditional virus–host model depicting a binary interaction in which Epstein–Barr virus (EBV) infects host cells with limited regulatory inputs. The right side shows the upgraded tripartite “Microbiome–Virus–Host axis” model illustrating the microbiome as an integrated ecological entity composed of bacteria, fungi, and microbial metabolites. The microbiome actively interacts with both EBV and host cells through bidirectional signaling. Four major functional modules are highlighted: regulation of viral latency, induction of reactivation, shaping of the immune microenvironment, and promotion of genomic instability. These interconnected processes converge to drive tumorigenesis, represented by cancer development at the bottom.

To address these questions, we propose the “microbiome–EBV axis” as a central analytical framework (**Figure 1**). We examine how commensal microorganisms may function as ecological regulators of viral carcinogenesis. This framework provides a potential explanation for the epidemiological patterns described above. To contextualize this concept, the following sections summarize the epidemiological landscape, major pathological subtypes, and global burden of EBV-associated cancers. This overview not only defines the clinical scale of these diseases but also highlights their distinct geographical distribution. Such patterns suggest that environmental and ecological factors, including region-specific microbial communities, may contribute to tumorigenesis beyond viral genetics and host susceptibility alone. This provides the rationale for considering the microbiome as a key variable.

Previous reviews have primarily examined EBV oncogenesis from a virus–host perspective (1). Separate reviews have addressed the broader role of the microbiome in human cancer (2). Other reviews have examined microbiome alterations in gastric cancer (3). Related work has also addressed microbiome–EBV interactions in nasopharyngeal cancer (4). In contrast, the present review provides an integrated and mechanism-oriented synthesis of the microbiome–EBV axis. Specifically, we organize the available evidence around the microbiome–EBV axis rather than treating microbial dysbiosis as a secondary association. This review differs from earlier work in three major aspects: first, it links microbial regulation of EBV latency and lytic reactivation with immune remodeling and genomic instability within a unified framework; second, it distinguishes reactivation-dependent mechanisms from reactivation-independent microbial mutagenesis; and third, it connects these mechanistic insights to translational issues, including biomarker discovery, microbiome modulation, and personalized therapeutic strategies. Thus, the unique contribution of this review is to synthesize microbiome–virus–host interactions into a systems-level framework for understanding why EBV-associated cancers arise only in specific ecological and clinical contexts.

To avoid overinterpreting the available evidence, this review explicitly distinguishes among different levels of support. Mechanistic or preclinical evidence refers to findings derived from cell-culture systems, organoids, animal models, or germ-free/humanized mouse models. Observational evidence refers to associations identified in patient cohorts, microbiome sequencing studies, tumor tissue analyses, or cross-sectional clinical datasets. Clinically validated evidence refers to findings supported by prospective clinical studies, interventional trials, or established clinical practice. Throughout this review, conclusions are therefore framed according to the strength of the available evidence, and microbiome-based diagnostic or therapeutic implications are discussed as emerging translational opportunities unless they have been clinically validated.

### 1.1 Literature search strategy

This narrative review was based on literature published between 1971 and July 2026 and identified through searches of PubMed, Web of Science, and Google Scholar. Search terms included combinations of “Epstein–Barr virus” or “EBV”, “microbiome” or “microbiota”, “carcinogenesis”, “viral latency”, “lytic reactivation”, “immune microenvironment”, “genomic instability”, “biomarker”, and “microbiome-targeted therapy”. Relevant original research articles, reviews, and clinical studies published in English were considered. Additional publications were identified by screening the reference lists of key articles. Because this article is a narrative review, no formal systematic review or meta-analytic selection procedure was applied.

### 1.2 Overview of EBV-associated cancers and their global burden

Epstein–Barr virus (EBV) is a ubiquitous human herpesvirus that establishes lifelong latency in more than 95% of adults worldwide. Taxonomically, it belongs to the *Lymphocryptovirus* genus of the *Herpesviridae* family, also designated human herpesvirus 4 (HHV-4). Its double-stranded DNA genome persists as a circular episome during latency, converting to a linear conformation upon lytic reactivation. Spanning approximately 175 kB, the genome encodes over 100 open reading frames and more than 40 microRNAs (1). EBV was the first human tumor virus to be identified (1). EBV is associated with malignancies of lymphoid, epithelial, and mesenchymal origin (5). Oncogenic mechanisms primarily stem from the hijacking of host signaling cascades by viral oncoproteins, notably Epstein-Barr nuclear antigen 1 (EBNA1) and latent membrane protein 1 (LMP1). LMP1 constitutively activates nuclear factor kappa B (NF-κB) and phosphatidylinositol 3-kinase/protein kinase B (PI3K/AKT) pathways, driving proliferative signaling while suppressing apoptosis. Concurrently, EBNA1 induces genomic instability by perturbing DNA repair machinery (5). These oncogenic effects are strongly dependent on the infected cell type. Although EBV infects most adults, malignant transformation occurs only in specific cellular, immunological, and environmental contexts (6). Taken together, these features indicate that EBV is a ubiquitous but context-dependent oncogenic agent whose malignant potential is only realized under specific cellular and ecological conditions.

In B lymphocytes, EBV infection is a primary driver of Burkitt lymphoma (BL) and Hodgkin lymphoma (HL). BL represents a highly aggressive non-Hodgkin lymphoma (NHL), frequently manifesting as extranodal masses or acute leukemia. Epidemiologically and clinically, BL stratifies into three distinct entities: endemic, sporadic, and immunodeficiency-associated. Endemic BL clusters in equatorial Africa; here, EBV seropositivity approaches 100%, with annual incidence rates reaching 5–10 per 100,000 in malaria-endemic zones (7). Repeated malaria exposure contributes to endemic Burkitt lymphoma by promoting B-cell activation and impairing EBV-specific immune control (8). Sporadic BL lacks specific geographic predilection, whereas immunodeficiency-associated BL predominates in HIV-positive cohorts. In developed nations, HL incidence stands at roughly 2–3 per 100,000 annually (9). Interestingly, higher socioeconomic status correlates with elevated risk, and incidence has increased in some populations (10). Etiological studies link EBV to approximately 25–40% of HL cases. Furthermore, genome-wide association studies (GWAS) validate EBV status as a critical etiological classifier for HL (11). These observations suggest that even within lymphoid malignancies, EBV-associated oncogenesis is influenced by the surrounding immunological and epidemiological context.

**Figure 2:**
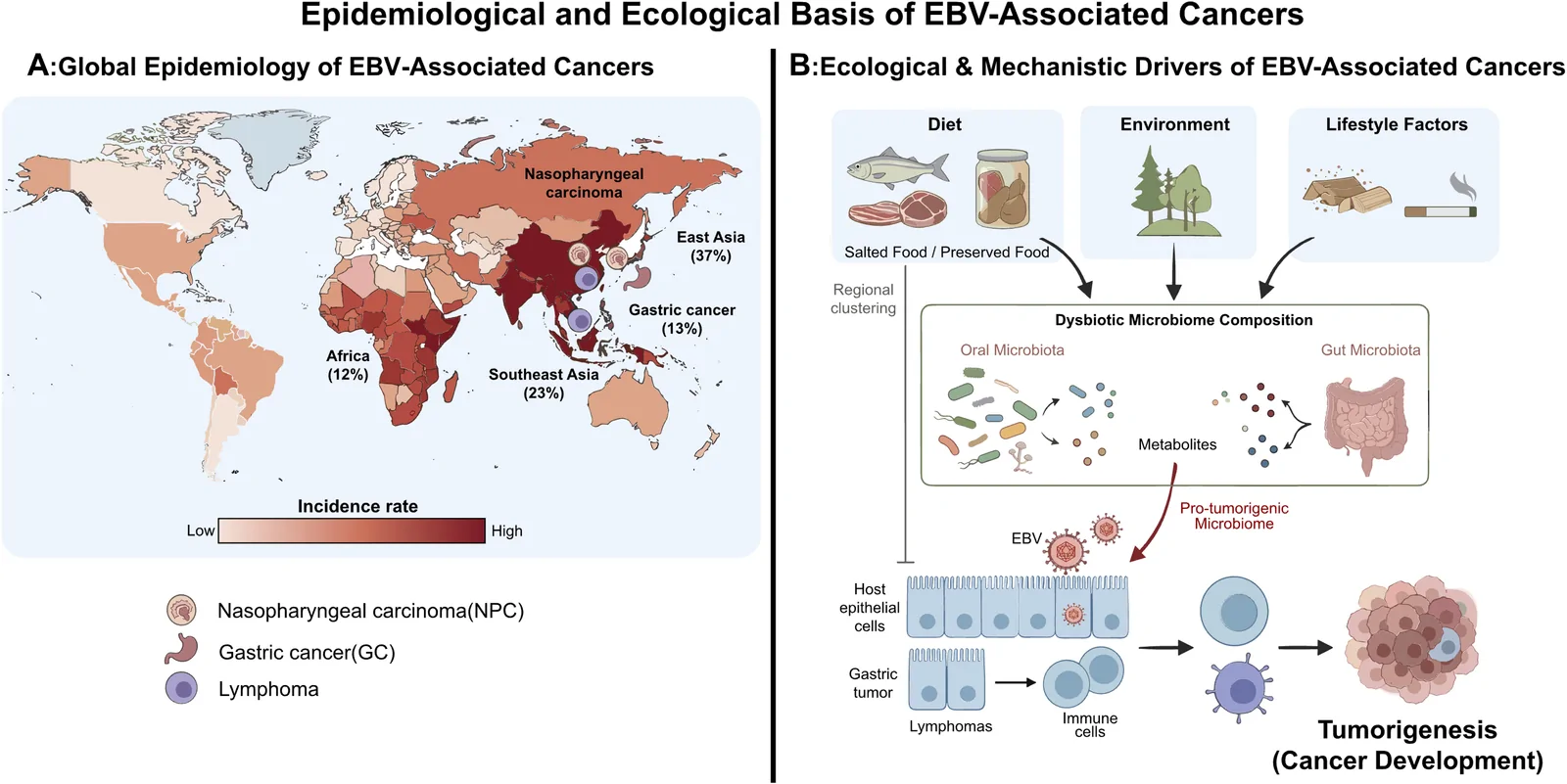
Epidemiological distribution and ecological determinants of EBV-associated cancers. **(A)** Global map showing the geographic distribution and incidence patterns of EBV-associated malignancies, including nasopharyngeal carcinoma, gastric cancer, and lymphoma, with higher prevalence in East Asia, Southeast Asia, and parts of Africa. **(B)** Conceptual model illustrating how environmental, dietary, and lifestyle factors shape regional microbiome composition in the oral and gut niches, leading to a pro-tumorigenic microbial ecosystem. This microbiome actively interacts with EBV and host epithelial or immune cells through metabolic and signaling pathways, forming a microbiome–virus–host axis. These interactions provide a mechanistic explanation for regional clustering and contribute to EBV-driven tumorigenesis.

Within the epithelial compartment, EBV targets nasopharyngeal and gastric mucosa via the Eph receptor A2 (EphA2) receptor. This tropism underpins the oncogenesis of EBV-positive nasopharyngeal carcinoma (NPC) and gastric carcinoma (GC) (12). NPC, an epithelial malignancy of the nasopharynx, shows tight coupling with EBV infection (13). While global annual incidence averages ~1 per 100,000, distribution exhibits marked geographic clustering. Southern China, Taiwan, and Southeast Asia collectively account for 80% of the global burden. NPC pathogenesis emerges from multifactorial interplay involving EBNA1-mediated viral genome maintenance, specific human leukocyte antigen (HLA)-linked host susceptibility, and environmental carcinogens such as nitrosamines derived from salted fish (14). GC ranks as the fifth most prevalent malignancy and the fourth leading cause of cancer mortality worldwide (15). Incidence peaks in Asian populations, particularly Japan and Korea (~41 per 100,000) (16). Emerging data confirm that a distinct subset of gastric cancers associates with EBV infection. This EBV-positive GC subtype harbors unique genomic aberrations, distinctive clinicopathological profiles, and confers a relatively favorable prognosis (17). Global statistics indicate over 200,000 new EBV-associated malignancies annually. NPC and GC comprise approximately 80% of these cases. Related mortality reaches 140,000 deaths, representing 2% of total global cancer mortality. Crucially, over 80% of this disease burden concentrates in low- and middle-income countries and regions (**Figure 2**). This geographic and socioeconomic concentration underscores the public health importance of EBV-associated malignancies (18). Such marked geographical concentration strongly argues that EBV-positive epithelial cancers arise from a multilayered interaction between viral persistence and region-specific environmental cofactors.

### 1.3 The microbiome as a regulator of viral persistence and tumorigenesis

The human microbiome, especially microbial communities colonizing mucosal barriers such as the gut and oropharynx, is increasingly recognized as a pivotal environmental modulator of persistent viral infection and carcinogenesis. Its function extends well beyond traditional roles in digestion and metabolic support (19). Through intricate mechanisms, the microbiome governs the dynamic equilibrium between EBV latency and lytic reactivation, while also shaping the host immune microenvironment and tumor-promoting tissue conditions. These broad processes are discussed in detail in the following mechanistic chapters.

Accordingly, the microbiome–EBV axis is used here as a framework for integrating microbial, viral, and host tissue–immune interactions during EBV-associated carcinogenesis. Representative microbial species and metabolites, their associated EBV-related pathways, proposed mechanisms, evidence levels, and potential clinical implications are summarized in **Table 1**.

**Table 1:** Representative microbial factors and their proposed roles in the microbiome–EBV axis.

Microbial species/factor	Main metabolite or component	Affected EBV-related pathway	Proposed mechanism	Supporting evidence	Potential clinical implication	Representative references
*Prevotella intermedia*	Cell wall components	EBV latency–lytic switch; BZLF1 activation	Engages TLR4 and activates MAPK signaling, promoting transcription factor binding at the BZLF1 promoter and facilitating EBV lytic reactivation.	Primarily preclinical mechanistic evidence.	Potential microbial trigger of EBV reactivation in oral or periodontal inflammatory niches; candidate target for oral microbiome risk modulation.	(20)
*Fusobacterium nucleatum*	Bacterial surface components and inflammatory signals	TLR/NF-κB signaling; EBV reactivation; inflammatory tumor microenvironment	Activates TLR/NF-κB signaling and may cooperate with EBV-driven inflammatory programs, thereby promoting a reactivation-prone and tumor-supportive niche.	Preclinical and observational evidence; stronger evidence in colorectal and oral cancer contexts than in EBV-specific causal models.	Candidate microbial biomarker and potential target for microbiome modulation, especially in mucosal EBV-associated tumors.	(21)
*Helicobacter pylori*	CagA, outer membrane proteins, inflammatory mediators	Chronic inflammation; NF-κB activation; AID induction; genomic instability	Promotes gastric inflammation and may cooperate with EBV infection by enhancing inflammatory signaling, DNA damage responses, and aberrant AID expression.	Observational clinical evidence and preclinical mechanistic evidence; EBV-specific causal evidence remains incomplete.	Potential cofactor in EBV-positive gastric cancer; supports risk stratification and possible relevance of H. pylori eradication in high-risk gastric cancer settings.	(22)
*Plasmodium falciparum*	Malaria antigens and chronic immune stimulation	EBV viral-load control; B-cell activation; AID-mediated mutagenesis; MYC–IGH translocation	Repeated malaria exposure promotes chronic B-cell activation, impairs EBV-specific immune surveillance, and increases the opportunity for AID-dependent genomic lesions.	Strong epidemiological association with endemic Burkitt lymphoma; supported by mechanistic and animal studies.	Supports antimalarial prevention strategies in endemic regions and EBV viral-load monitoring in high-risk populations.	(23)
*Aggregatibacter actinomycetemcomitans* and other CDT-producing bacteria	Cytolethal distending toxin (CDT), especially CdtB	DNA damage response; EBV lytic reactivation; genomic instability	CDT induces DNA double-strand breaks and activates DNA damage responses, which may destabilize EBV latency and increase genomic instability in EBV-infected epithelial cells.	Strong preclinical evidence, including EBV-infected epithelial-cell models; limited direct clinical validation.	Potential microbial genotoxic risk factor and candidate target for oral or gastric microbiome surveillance.	(24)
SCFA-producing commensals	Butyrate, acetate, propionate	BZLF1 promoter regulation; HDAC-dependent chromatin remodeling; Treg/Th17 balance	SCFAs, especially butyrate, inhibit HDAC activity, alter viral and host chromatin accessibility, and modulate immune-cell differentiation.	Strong preclinical evidence for epigenetic regulation; clinical relevance remains context-dependent.	Possible metabolite biomarker and therapeutic modulator; requires caution because butyrate may regulate immunity while also inducing EBV lytic activation.	(25)
*Porphyromonas gingivalis*	Butyrate and periodontal inflammatory products	EBV lytic reactivation; LMP1 expression; local inflammatory signaling	Periodontal dysbiosis may provide inflammatory and epigenetic cues that facilitate EBV reactivation or alter latent gene expression.	Mainly observational and *in vitro* evidence.	Potential oral microbiome biomarker or intervention target in nasopharyngeal and oral EBV-related disease contexts.	(26)
*Gemella* spp. and *Streptococcus sanguinis*	Hydrogen peroxide and oral microbial metabolites	ROS-associated EBV lytic reactivation; genomic instability	Oral microbial metabolites may increase oxidative stress, promote EBV lytic activation, and contribute to DNA damage in susceptible tissues.	Observational association in NPC-related oral microbiome studies, with mechanistic plausibility from ROS studies.	Potential oral microbial markers for EBV reactivation risk and NPC susceptibility.	(27)
Gram-negative dysbiosis	Lipopolysaccharide (LPS)	TLR4/NF-κB/MAPK signaling; EBV lytic reactivation	Barrier dysfunction may increase systemic or local LPS exposure, leading to sustained TLR4 signaling and inflammatory mediators that lower the threshold for EBV reactivation.	Preclinical and mechanistic evidence; clinical relevance inferred from dysbiosis and inflammatory states.	Supports barrier-protective and anti-inflammatory microbiome strategies; LPS-related signatures may help identify inflammatory reactivation-prone states.	(28)
Gut microbiota associated with immune checkpoint response	SCFAs, indole derivatives, bile acid metabolites	PD-L1/CTLA-4 signaling; CD8^+^ T-cell exhaustion; NK-cell function	Microbial metabolites and immune priming effects can modulate checkpoint expression, T-cell differentiation, and cytotoxic lymphocyte function, thereby influencing immune escape.	Clinical and experimental evidence in broader cancer immunotherapy; EBV-specific evidence remains partly indirect.	Potential adjuvant strategy for checkpoint blockade or cellular therapy in EBV-associated malignancies.	(29)
Probiotics, including Bifidobacterium and Lactobacillus species	Extracellular vesicles, SCFAs, indole derivatives	Inflammatory signaling; antiviral immunity; immune checkpoint responsiveness	May restore barrier integrity, regulate dendritic-cell activity, enhance cytotoxic T-cell responses, and reduce inflammatory cues that promote EBV reactivation.	Mostly preclinical or extrapolated from non-EBV cancer immunotherapy studies.	Possible adjunctive microbiome-modulating strategy, but EBV-specific clinical validation is still limited.	(30)
Fecal microbiota transplantation or defined microbial consortia	Whole microbial communities and functional microbial metabolites	Systemic immune homeostasis; checkpoint response; EBV-related immune surveillance	May remodel gut microbial ecology and restore antitumor immune competence, potentially improving immune control of EBV-associated tumors.	Clinical evidence exists in broader cancer immunotherapy settings; direct EBV-associated cancer evidence remains limited.	Potential future strategy for immune restoration, but safety, donor variability, colonization stability, and EBV reactivation risk require monitoring.	(31)

Evidence levels are summarized as preclinical evidence, observational evidence, or clinically supported evidence. Preclinical evidence includes *in vitro*, organoid, animal, and germ-free or humanized mouse studies. Observational evidence includes patient cohort, sequencing, tissue profiling, and cross-sectional clinical studies. Clinically supported evidence refers to findings supported by prospective clinical studies, interventional studies, or established clinical practice. Most microbiome-based interventions in EBV-associated cancers remain preclinical or early translational.

## 2 Microbial triggers of EBV reactivation

**Figure 3:**
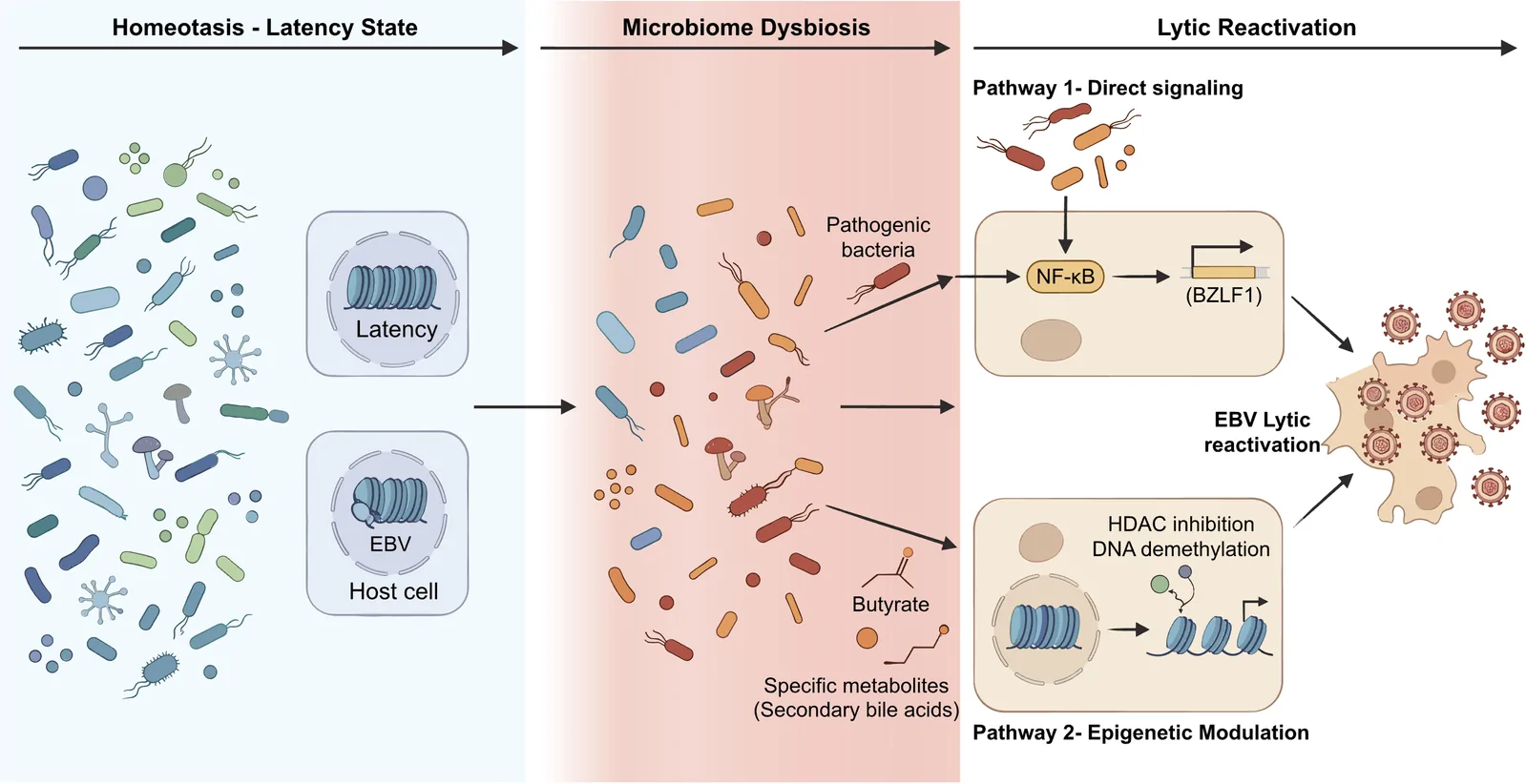
Microbiome regulation of the EBV latency–lytic switch. Schematic illustrating the dynamic transition from EBV latency to lytic reactivation under microbiome influence. A balanced microbiome maintains viral latency by preserving a condensed chromatin state and limiting transcriptional activation. Microbiome dysbiosis, characterized by reduced diversity and expansion of pathogenic taxa, promotes reactivation through two parallel mechanisms: direct activation of host signaling pathways such as NF-κB that induce immediate-early genes such as BZLF1, and epigenetic modulation by microbial metabolites such as butyrate and secondary bile acids that alter chromatin accessibility via HDAC inhibition and DNA demethylation. These pathways converge to drive viral replication and lytic cycle progression.

The microbiome may contribute to EBV-associated carcinogenesis by modulating the viral life-cycle switch between latency and lytic reactivation. Latency supports persistent infection and immune evasion, whereas the lytic phase contributes to viral transmission, inflammation, and genomic instability (1). Specific microbes or their metabolites may function as context-dependent triggers of this transition. Rather than acting solely through nonspecific inflammation, these factors may influence EBV latency maintenance by mimicking or amplifying endogenous host signals (32) (**Figure 3**). To examine this mechanism, we first consider direct ligand–receptor interactions. Microbial structural components can engage pattern-recognition and B-cell receptor-associated signaling pathways, thereby activating downstream signaling and EBV immediate-early regulators such as BZLF1 and BRLF1 (33). This positions the latency-to-lytic transition as a mechanistic bottleneck through which microbial perturbation may contribute to EBV-associated carcinogenic processes.

### 2.1 Pathogens as reactivation catalysts

#### 2.1.1 The roles of Helicobacter pylori, Plasmodium falciparum, and bacterial lipopolysaccharide in TLR-mediated EBV reactivation

The interplay between chronic microbial colonization and dysregulated host immunity constitutes a critical nexus for EBV reactivation from latency. Accumulating evidence implicates specific pathogens and their constituents—particularly *Helicobacter pylori* and bacterial LPS—in perturbing Toll-like receptor (TLR) signaling axes, thereby disrupting EBV latency.

Chronic *H. pylori* infection is a major risk factor for gastric inflammation and carcinogenesis (34). Notably, *H. pylori* colonization and EBV latent infection can coexist within the gastric mucosa (35). A recent analysis of gastritis, intestinal metaplasia, and gastric cancer specimens detected EBV/H. pylori coinfection in both intestinal metaplasia and gastric cancer tissues. H. pylori-positive samples also showed increased expression of the EBV immediate-early lytic gene BZLF1 (36). These findings suggest that H. pylori-associated inflammation or specific virulence factors may create conditions that favor EBV persistence or lytic reactivation, although direct causality remains unestablished. Cytotoxin-associated gene A (CagA), a major *H. pylori* virulence factor, activates oncogenic YAP signaling and promotes epithelial–mesenchymal transition in gastric epithelial cells (37). In asymptomatic young adults, *H. pylori* eradication was reported to restore rather than disrupt the gastrointestinal microbiota (38). These findings suggest that *H. pylori* eradication may help normalize gastrointestinal microbial composition. TLR signaling also contributes to mucosal immune responses during *H. pylori* infection (39). Additionally, H. pylori adhesins such as BabA facilitate bacterial adherence to gastric mucins and contribute to persistent colonization (40). Their mechanisms likely involve engagement with pattern recognition receptors, including TLRs (39). Collectively, these findings support the view that chronic bacterial colonization creates a permissive inflammatory niche in which EBV latency becomes increasingly unstable.

Lipopolysaccharide (LPS), a major component of the Gram-negative bacterial outer membrane and a canonical TLR4 ligand, provides another route by which dysbiosis may destabilize EBV latency (41). Gut barrier disruption can facilitate systemic exposure to immunostimulatory LPS, resulting in sustained TLR4 engagement and activation of inflammatory NF-κB/MAPK signaling (42). Accordingly, microbiota-derived endotoxemia may lower the threshold for EBV reactivation by sustaining TLR4-dependent inflammatory signaling (28).

#### 2.1.2 Fungal metabolites and epigenetic regulation of EBV lytic genes

*Aspergillus*-derived secondary metabolites, particularly aflatoxin B_1_ (AFB1) (43), may influence the EBV latency–lytic switch by altering DNA methylation and histone modification (44). AFB1 exposure has been associated with changes in DNA methylation, including alterations involving EBV regulatory regions (44). DNA methylation of CpG-rich regulatory regions contributes to transcriptional silencing of EBV lytic genes (45). DNA methyltransferases (DNMTs), notably DNMT3B, contribute to maintenance of the EBV methylation landscape. Conversely, DNMT inhibitors like 5-azacytidine can promote promoter demethylation and restore viral transcriptional competence (46). Murine oocyte models exposed to AFB1 exhibit either elevated methylation or global hypomethylation (47). Human data parallel these findings, linking AFB1 exposure to genome-wide hypomethylation, especially in hepatocellular carcinoma cohorts (48). Such epigenetic disequilibrium directly disrupts the EBV latency–lytic switch. Epigenetic relaxation of the BZLF1 (encoding Zta) and BRLF1 (encoding Rta) promoters can facilitate the expression of EBV immediate-early lytic genes (46). Accardi et al. reported that AFB1 activated the EBV lytic cycle, increased viral load, and promoted EBV-driven B-cell transformation in experimental models (49).

AFB1 perturbs host metabolic flux, starving the epigenetic machinery of essential substrates and driving aberrant methylation within EBV lytic promoters. Specifically, by depleting S-adenosylmethionine (SAM) binding activity (50), AFB1 induces hypomethylation at key loci like BZLF1 and BRLF1 (51), effectively triggering viral reactivation. Beyond DNA methylation, histone modifications also contribute to the regulation of EBV lytic gene expression (44). In latent infection, repressive marks such as H3K27me3 and H4K20me3 silence the BZLF1 promoter (49). However, challenging cells with histone deacetylase inhibitors such as TSA or specific methyltransferase inhibitors such as DZNep sharply elevates BZLF1 transcripts (52). Chaetocin represents a mechanistically distinct example: it promotes EBV lytic reactivation primarily through ROS-dependent signaling, and this effect can be inhibited by N-acetylcysteine(NAC) (53).Overall, fungal metabolites may influence EBV transcription through epigenetic and oxidative-stress-related mechanisms, although the direct links among AFB1 exposure, specific viral chromatin alterations, and lytic reactivation require further validation.

### 2.2 Mechanistic pathways

#### 2.2.1 ROS generation and oxidative stress

Reactive oxygen species (ROS)-mediated oxidative stress serves as a pivotal environmental trigger for microbial-induced EBV lytic reactivation. Early work established that EBV infection per se disrupts cellular redox homeostasis. *In vitro* assays confirmed that infection elevates malondialdehyde (MDA), a lipid peroxidation marker, while concurrently suppressing catalase and superoxide dismutase (SOD) activities (54). These data provide the first direct evidence of a global oxidative stress signature induced by EBV in host cells. Subsequent investigations revealed this signature is not merely a passive sequel of infection; rather, it is actively orchestrated by specific EBV-encoded products.

Accumulating evidence indicates EBV remodels host redox equilibrium to foster a microenvironment conducive to lytic replication. In nasopharyngeal carcinoma models, Hu et al. demonstrated that the EBV oncoprotein LMP1 drives a “redox reset” (55). This process involves upregulating NADPH oxidase (NOX) isoforms NOX1 and NOX2 while activating the core antioxidant regulator, nuclear factor erythroid 2-related factor 2 (Nrf2). The outcome is a distinct host cell redox state: high ROS accumulation coexists with robust antioxidant capacity. Such an environment not only sustains EBV latency but also primes conditions for lytic reactivation (55). Huang et al. showed that N-methyl-N’-nitro-N-nitrosoguanidine (MNNG) induces EBV entry into the lytic cycle in a dose-dependent fashion (56). Treatment with 1 µg/mL MNNG for 72 hours drove over 70% of NA cells into the lytic phase. MNNG exposure markedly spikes intracellular ROS levels. Crucially, the ROS scavenger N-acetyl-L-cysteine (NAC) abrogates MNNG-induced reactivation. Similarly, H_2_O_2_ triggers EBV reactivation, a process likewise inhibited by NAC (56). Chaetocin, a fungal metabolite exhibiting antibacterial and cytostatic properties (57), significantly upregulates EBV lytic transcription and DNA replication even at low concentrations (50 nM). Latent EBV activation coincides with elevated cellular ROS, and NAC effectively blocks chaetocin-induced activation. While chaetocin minimally impacts histone H3K9 methylation, NAC substantially reduces H3K9 methylation levels. These findings suggest chaetocin primarily reactivates latent EBV via the ROS axis (58). Together, these studies indicate that intracellular ROS promotes EBV lytic reactivation, whereas antioxidant treatment suppresses this process.

Multiple EBV-encoded proteins synergistically amplify oxidative stress signals. Beyond LMP1, EBNA1 induces ROS generation through NOX pathway activation, and EBERs have also been implicated in oxidative stress responses (58). This virus-driven oxidative milieu finds corroboration in clinical specimens. Nasopharyngeal carcinoma biopsies exhibit significant elevation of oxidative stress markers like inducible nitric oxide synthase (iNOS). Furthermore, patient serum levels of the DNA oxidative damage marker 8-hydroxy-2’-deoxyguanosine (8-OHdG) show a strong positive correlation (r=0.72, p<0.001) with the EBV lytic reactivation marker Rta-IgG (59). Notably, Michael Y. Bonner posited that EBV-positive tumors are essentially “ROS-driven tumors”. Here, viral lytic reactivation and ROS generation form a self-amplifying loop in which EBV-encoded proteins such as LMP1 stimulate ROS production, and accumulated ROS further propels the viral transition from latency to the lytic phase. Ultimately, this accelerates genomic instability and carcinogenesis (60). This reciprocal reinforcement between oxidative stress and viral activation helps explain why ROS constitutes a central bridge linking microbial stimuli to EBV-driven carcinogenesis.

Targeting oxidative stress pathways represents a promising therapeutic avenue for EBV-driven malignancies. Preclinical data demonstrate that natural antioxidants effectively suppress EBV lytic reactivation. Resveratrol disrupts transcriptional initiation of EBV immediate-early genes by preventing transcription factor binding to the BRLF1 and BZLF1 promoters (61). Epigallocatechin gallate (EGCG) has been reported to modulate LMP1-associated signaling and oxidative-stress pathways in EBV-related models (62). Together, these antioxidant studies support the view that redox balance can modulate EBV lytic reactivation. However, most evidence remains preclinical, and the therapeutic value of antioxidant-based modulation in EBV-associated cancers requires further validation. Future investigations should probe the synergy between antioxidant therapies and standard chemoradiotherapy to devise precision interventions for EBV-associated cancers.

#### 2.2.2 Microbiota-induced B cell activation

EBV exploits host epigenetic machinery to restrict its own gene expression, establishing lifelong latency within memory B cells and evading immune surveillance (63). This latent state remains dynamically governed by host-microbe crosstalk, particularly via B cell receptor (BCR) and Toll-like receptor (TLR) signaling cascades. Commensal-derived microbial antigens such as lipopolysaccharide (LPS) engage B cell TLRs including TLR2, TLR4, and TLR9. This engagement triggers downstream NF-κB and MAPK signaling (64). NF-κB signaling can induce DNMT3B-dependent DNA methylation changes in EBV-infected cells (65). DNA methylation is also an important regulator of EBV transcriptional states and latency (46). TLR activation can also induce interferon-stimulated gene 15 (ISG15) expression in CD8^+^ T cells (66). In EBV-positive gastric cancer, ISG15^+^CD8^+^ T cells display a precursor exhausted phenotype yet retain proliferative potential; their frequency correlates with robust anti-EBV immunity (67). Crucially, TLR agonists like CpG stimulate proliferation in EBV-infected B cells, indicating that these agonists reshape both metabolism and gene expression profiles. Metabolic rewiring may reverse repressive histone methylation marks on the viral genome, consequently altering latent membrane protein (LMP) expression and lytic cycle gene transcription (51). Accordingly, microbiota-induced B-cell activation is likely to influence EBV persistence not only through inflammatory signaling, but also through durable rewiring of the epigenetic landscape.

BCR engagement and TLR stimulation can cooperate to lower the threshold for B-cell activation (68). Upon binding microbial antigens, the BCR triggers internalization and downstream kinase cascades involving Lyn and Syk. These events assemble a “signalosome” that optimizes signaling efficiency (69). Concurrently, TLR ligands such as bacterial CpG DNA or LPS engage the myeloid differentiation primary response 88 (MyD88)-dependent axis. This pathway cooperates with BCR signaling to lower the activation threshold. Mechanistically, TLR ligation activates cofilin, an actin-severing protein, thereby remodeling the cytoskeleton. Such restructuring reduces the spatial confinement of BCRs within the plasma membrane. Consequently, increased receptor mobility facilitates frequent collisions and robust signal transduction, even under conditions of low antigen density (68). This dual signaling architecture drives upregulation of latent membrane protein 1 (LMP1) via the non-canonical NF-κB (RelB/p52) pathway (70). As an EBV-encoded constitutively active oncoprotein, LMP1 perpetually engages NF-κB and AP-1 signaling axes. This drives B cell proliferation and survival (71), ultimately establishing a microenvironment permissive for viral reactivation.

Microbial signal integration extends beyond immediate activation to induce long-term epigenetic reprogramming. Repeated co-stimulation of BCR and TLR upregulates interferon regulatory factor 4 (IRF4). This transcription factor progressively opens chromatin domains associated with plasma cell differentiation. Such “epigenetic imprinting” accumulates with successive antigen exposures, skewing the BLIMP1-to-BACH2 expression ratio (BLIBA) in memory B cells. While BLIMP1 commits cells to plasma cell differentiation, BACH2 supports germinal center re-entry. An elevated BLIBA biases lineage commitment toward the plasma cell fate (72). This shift is critical for EBV reactivation, as viral lytic genes like BRLF1 are preferentially transcribed in differentiated plasma cells. Notably, microbial adjuvants such as CpG (a TLR9 ligand) can be engineered into nanovaccines co-delivering BCR antigens and TLR ligands, exploiting this synergistic potential. For instance, CpG-conjugated tumor membrane vesicles drive robust B cell activation through BCR clustering and TLR9 co-stimulation, enhancing antigen presentation and downstream signaling (73). This combinatorial transduction mirrors mechanisms by which ligands derived from gut or oral microbiota, including *Gemella* and *Parvimonas*, may reactivate EBV in mucosal-infiltrating B cells, thereby linking commensal dysbiosis to viral oncogenesis.

### 2.3 Microbial regulation of the EBV latency barrier

Collectively, current evidence supports microbial regulation of the EBV latency–lytic switch through receptor-mediated signaling and epigenetic remodeling, although the clinical relevance of these mechanisms remains incompletely established.

In addition to reactivation-associated genomic injury, microorganisms may contribute to genomic instability through reactivation-independent mutagenic mechanisms, which are discussed separately in Chapter 4.

Direct mechanistic evidence is derived mainly from controlled experimental studies, including work showing that bacterial genotoxins can trigger EBV reactivation and genomic instability in infected epithelial cells (24). Observational clinical support has been reported in nasopharyngeal carcinoma cohorts (32). Related microbial alterations have also been described in EBV-positive gastric cancer (74). The strongest mechanistic support comes from *in vitro* studies showing that bacterial products, microbial metabolites, or genotoxins can activate EBV immediate-early genes such as BZLF1 and BRLF1. These systems are valuable because they establish direct biological plausibility under controlled conditions. However, they may not fully reproduce the spatial organization, immune pressure, metabolite gradients, and microbial community complexity of EBV-associated tumor tissues. By contrast, clinical studies linking dysbiosis to EBV load or EBV-positive tumor status provide important disease relevance, but most are cross-sectional and therefore cannot determine whether microbial alterations drive EBV reactivation or arise secondarily from tumor-associated ecological changes. Thus, microbial regulation of EBV reactivation should currently be interpreted as a plausible and experimentally supported mechanism, but one that still requires longitudinal and *in vivo* validation.

## 3 Microbiome-associated regulation of host immunity in EBV pathogenesis

**Figure 4:**
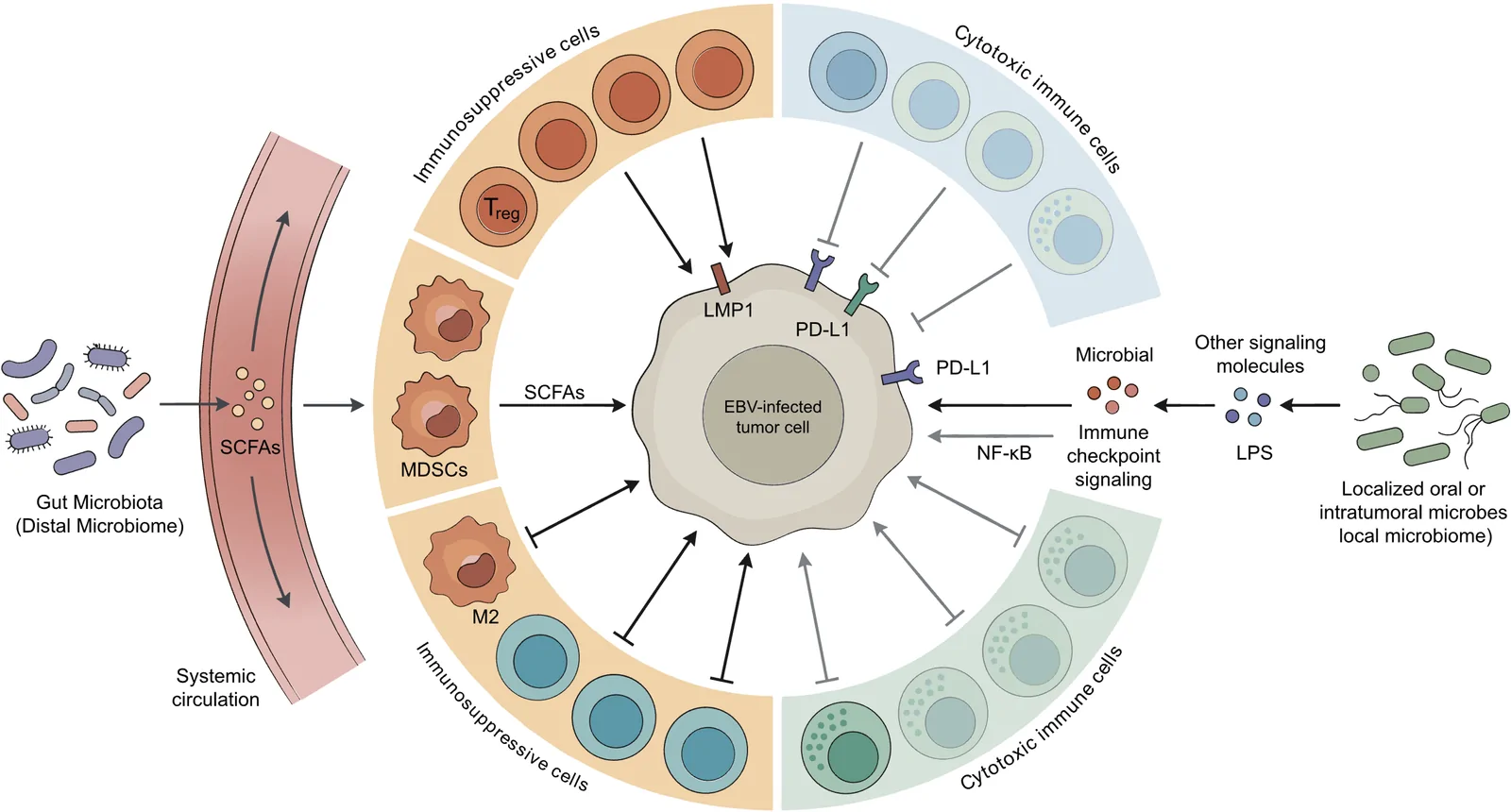
Microbiome-driven shaping of an immunosuppressive tumor microenvironment in EBV-associated cancers. Schematic illustrating how distal and local microbiota cooperatively remodel the tumor immune microenvironment. EBV-infected tumor cells expressing viral proteins such as LMP1 and immune checkpoint molecules such as PD-L1 are surrounded by key immune populations. Microbiome-derived signals from the gut, such as short-chain fatty acids, together with products released by local microbes, such as LPS, promote the expansion and polarization of immunosuppressive cells, including regulatory T cells (Tregs), myeloid-derived suppressor cells (MDSCs), and M2 macrophages. Concurrently, these signals inhibit the cytotoxic functions of CD8^+^ T cells and natural killer (NK) cells and enhance immune checkpoint signaling. Together, these effects establish an immunosuppressive niche that facilitates immune evasion and supports EBV-driven tumor progression.

Microbial control of Epstein-Barr virus (EBV) extends far beyond the mere induction of lytic replication. A more profound, clinically salient mechanism involves the microbiome’s capacity to systematically reconfigure the host immune landscape (**Figure 4**). This remodeling fosters an “immune-permissive”, if not overtly immunosuppressive, niche that abets the survival, clonal expansion, and malignant transformation of EBV-infected cells (2). Such modulation permeates the innate-to-adaptive immunity continuum, enabling local or distal dysbiosis to compromise tumor immune surveillance efficacy. Here, we examine how microbiome-associated factors may interact with EBV to influence key immune components of the tumor microenvironment (TME). Key targets include immune checkpoint upregulation, effector lymphocyte exhaustion, and the accrual of regulatory populations (75). The novelty of this paradigm lies in linking distal microbial ecosystems—specifically the gut—to local tumor immune status via metabolic crosstalk, signaling mediators, and cellular trafficking. Together, these elements constitute a systemic “gut-tumor axis” governing immune regulation (76). To navigate this complex network, we first examine microbial regulation of critical checkpoints: programmed death-ligand 1 (PD-L1) and cytotoxic T-lymphocyte-associated protein 4 (CTLA-4). We will delineate how microbial metabolites such as short-chain fatty acids or bacterial translocation modulate checkpoint expression on EBV-infected or stromal cells. These effects are mediated through epigenetic remodeling or signal transduction cascades (77). Ultimately, this reveals a mechanistic conduit by which the microbiome dictates immunotherapy responsiveness and facilitates viral immune evasion (78). This perspective recasts immune regulation as a central arena in which microbial ecology can dictate the clinical behavior of EBV-associated tumors.

### 3.1 Immune checkpoints and microbial modulation

#### 3.1.1 Gut microbiota modulates immune checkpoint signaling in EBV-associated malignancies

The gut microbiota may influence immune checkpoint activity in EBV-associated tumors, although direct EBV-specific evidence remains limited. Clinical data underscore a tight correlation between EBV infection and PD-L1 upregulation in gastric cancer tissues. PD-L1 overexpression has been documented in EBV-positive gastric cancer (79). In nasopharyngeal carcinoma, NF-κB/STAT3-associated PD-L1 expression also contributes to immune escape (80). Viral-encoded microRNAs likely mediate this regulation. For instance, EBV-miR-BART18-3p activates the SIRT1/HIF-1α/lactate dehydrogenase axis. This promotes lactate accumulation and histone acetylation within the TME (81). Confirmed studies show that such lactate buildup suppresses T cell activity through diverse mechanisms while concurrently driving PD-L1 transcription and surface expression (82). Specific commensals also engage directly in checkpoint modulation. *Bifidobacterium* augments antitumor immunity by refining dendritic cell function, thereby potentiating PD-L1 inhibitor efficacy (29). Meanwhile, the model strain *Coprobacillus cateniformis* recalibrates checkpoint pathway activity. It targets the PD-L2–RGMb signaling axis to heighten sensitivity to anti-PD-1 therapy (83). Together, these findings provide indirect support for microbiome-mediated regulation of immune checkpoint responses in EBV-associated tumors.

Gut dysbiosis further potentiates these effects via microbial metabolites, including short-chain fatty acids (SCFAs) and secondary bile acids. For instance, butyrate downregulates PD-L1 expression within the tumor microenvironment by attenuating STAT3 and NF-κB pathway phosphorylation (77). Indolepropionic acid (IPA), a co-metabolite of *Lactobacillus johnsonii* and *Clostridium sporogenes*, drives the differentiation of progenitor exhausted T cells (Tpex). It achieves this by enhancing H3K27 acetylation at the *Tcf7* super-enhancer in CD8^+^ T cells, thereby sensitizing melanoma and colorectal cancer models to anti-PD-1 blockade (84). In contrast, phenylacetylglutamine (PAGln) acts as a detrimental microbial metabolite that compromises anti-PD-1 therapeutic efficacy (85). The immunological outcome therefore depends on the balance between protective and deleterious microbial metabolites within the tumor-bearing host.

CTLA-4 modulation by the gut microbiota hinges largely on bacterial translocation to the tumor niche and the ensuing local immune activation. During CTLA-4 blockade with agents such as ipilimumab, dysbiotic shifts including *Bacteroides* depletion or *Proteobacteria* expansion compromise mucosal barrier integrity. This breach facilitates the translocation of bacterial components like lipopolysaccharide (LPS) or live bacteria into lymphoid tissues and tumor sites (86). Via pattern recognition receptors such as TLR2 and TLR4, these microbial antigens activate tumor-infiltrating myeloid subsets, including dendritic cells and macrophages (87). The result is a surge in pro-inflammatory cytokines, such as IL-6, TNF-α, and IL-12 (86). Such dysbiosis-driven inflammation alters immune cell functional states, indirectly tuning CTLA-4 expression on regulatory T cells (Tregs) and exhausted T cells, which ultimately suppresses effector T cell antitumor activity (88). In effect, dysbiosis-driven myeloid activation establishes a cytokine milieu that indirectly entrenches T-cell dysfunction.

Clinical data corroborate this: melanoma patients treated with anti-CTLA-4 agents such as ipilimumab exhibit superior outcomes when harboring high microbial diversity and enrichment of *Bacteroides* species including *B. fragilis* and *B. thetaiotaomicron* (88). Fecal microbiota transplantation (FMT) from responders—characterized by *Bacteroidales* abundance—into germ-free mice significantly augments CTLA-4 blockade efficacy, whereas FMT from non-responders yields no benefit (86). These findings underscore the pivotal role of microbiome in regulating the CTLA-4 axis.

Collectively, the gut microbiota and its metabolites dynamically orchestrate PD-L1/CTLA-4 expression in EBV-positive tumors through multifaceted mechanisms spanning epigenetic remodeling, signaling cascade activation, and virus-microbe crosstalk. Whether microbiome-targeted interventions improve responses to immune checkpoint inhibitors in EBV-associated malignancies requires direct clinical validation.

#### 3.1.2 Commensal-derived short-chain fatty acids modulate the Treg/Th17 axis

Gut commensals ferment dietary fiber to generate short-chain fatty acids (SCFAs), including acetate, propionate, and butyrate (89). These metabolites contribute to host immune homeostasis. Under physiological conditions, SCFAs directly influence the Treg/Th17 differentiation balance via epigenetic mechanisms and signaling pathways (90). Commensal-derived butyrate, for example, promotes Treg lineage commitment and function by inhibiting histone deacetylases (HDACs) (91). This inhibition increases histone acetylation at the *Foxp3* locus, thereby supporting immune tolerance and limiting excessive inflammation (75). SCFAs also regulate T-cell differentiation through receptor-dependent mechanisms. By engaging G protein-coupled receptors, particularly free fatty acid receptor 2 (FFAR2) and free fatty acid receptor 3 (FFAR3), SCFAs promote dendritic-cell secretion of TGF-β and retinoic acid, thereby promoting Treg differentiation. These metabolites also suppress RORγt activity and reduce Th17 differentiation (90). Restoration of SCFA production and intestinal homeostasis may reduce inflammatory signaling and help rebalance Th17/Treg responses (90). This immune homeostasis may be relevant during EBV infection, in which viral immune evasion and chronic inflammation may contribute to disruption of the Treg/Th17 balance.

Microbial dysbiosis can reduce SCFA availability, potentially disrupting the Treg/Th17 balance and contributing to disease progression. Clinical studies have reported an increased abundance of *Prevotella copri* and a reduced abundance of butyrate-producing taxa such as *Faecalibacterium* in patients with rheumatoid arthritis (RA). Reduced SCFA availability may promote Th17 expansion and IL-17A production, thereby contributing to synovial inflammation (92). In cohorts with EBV-associated hepatic dysfunction, the abundance of butyrate producers such as *Prevotella* and *Collinsella* correlates inversely with serum alanine aminotransferase (ALT) levels and CD8^+^ cytotoxic T lymphocyte infiltration. Conversely, Granulicatella and Acinetobacter abundance was positively associated with IL-1β, IL-6, and TNF-α levels (93). Experimental studies further support the role of microbiota-derived signals in Treg/Th17 regulation: fecal transplantation from RA donors induces a Th17/Treg imbalance and an arthritic phenotype. Supplementation with *Lactobacillus casei* CCFM1074 mitigates joint pathology by restoring SCFA levels, reducing Th17 responses, and expanding the Treg compartment (94). EBV infection itself can trigger gut microbiota perturbation, leading to immune checkpoint dysregulation via SCFA depletion. In mice challenged with EBV DNA, colonic IL-17A^+^/IFN-γ^+^ and IL-17A^+^/FOXP3^+^ T-cell populations were increased, suggesting altered T-cell polarization and Treg plasticity (95). This shift may be associated with LPS translocation following barrier disruption and subsequent activation of the IL-23/Th17 axis. These associations suggest that depletion of butyrate-producing commensals may reduce SCFA availability and promote inflammatory liver injury.

SCFA supplementation and microbiota-based approaches have been investigated as potential immunomodulatory strategies. However, their therapeutic relevance to EBV-associated malignancies remains to be established. Exogenous butyrate, for instance, suppresses the NF-κB axis via HDAC inhibition. This mechanism may promote Treg responses and reduce Th17 responses (96). In dextran sulfate sodium (DSS)-induced colitis models, Astragalus polysaccharide (APS) restores butyrate output in a microbiota-dependent fashion. It also upregulates FFAR2/FFAR3 expression, promotes Treg differentiation, and inhibits Th17 polarization, antibiotic-mediated microbiota depletion abolishes this effect (90). Similarly, Schisandra chinensis pollen extract modulates the Treg/Th17 balance by enriching *Akkermansia* and *Lactobacillus* and increasing SCFA production (97). As pivotal mediators of host-microbiota crosstalk, SCFAs rewire the Treg/Th17 balance through epigenetic remodeling and receptor signaling, thereby modulating the inflammatory and carcinogenic niches associated with EBV. Future inquiries must dissect the dynamic roles of SCFAs across the spectrum from EBV latency to malignant transformation, paving the way for precision immuno-interventions grounded in SCFA metabolic profiles.

### 3.2 Pathogen-mediated immune evasion

#### 3.2.1 Coinfection compromises EBV-specific CD8^+^ T cell surveillance

Within the tumor-promoting microenvironment of chronic Epstein-Barr virus (EBV) infection, virus-specific CD8^+^ T cells serve as pivotal effectors of immune surveillance. These cytotoxic lymphocytes recognize viral antigens—such as BZLF1 expressed during the lytic phase and EBNA3 proteins in latency, and execute target cell lysis via perforin and granzyme B, while concurrently secreting pro-inflammatory cytokines like interferon-γ (IFN-γ) (98). Yet, concurrent or secondary microbial challenges can subvert this defense through diverse mechanisms, facilitating EBV immune escape and hastening oncogenesis.

Coinfection-induced remodeling of the immune niche profoundly impairs EBV-specific CD8^+^ T cell functionality. Ordinarily, CD8^+^ T cell efficacy hinges on dendritic cell (DC) antigen presentation, co-stimulatory signaling, and local microenvironmental cues (99). Commensal microbiota modulate DC maturation via metabolites—notably short-chain fatty acids (SCFAs)—and pattern recognition receptor pathways, including Toll-like receptors. Certain taxa, such as *Ruminococcaceae* YB328, upregulate DC surface expression of CD80/CD86 and MHC class I molecules, boost IL-12p70 secretion, and thereby potentiate CD8^+^ T cell priming and expansion (100). In contrast, coinfection or dysbiosis disrupts this immunoregulatory axis. For instance, post-liver transplant pediatric patients receiving immunosuppressants exhibit gut dysbiosis strongly correlated with heightened EBV reactivation risk—a phenomenon linked to defective DC maturation and attenuated CD8^+^ T cell responses (101). Moreover, in EBV-driven malignancies such as nasopharyngeal carcinoma and gastric cancer, the intestinal microbiome actively fosters viral persistence and tumorigenesis. Angela Wahl et al. demonstrated using germ-free humanized mice that conventionally colonized (CV) animals, unlike germ-free (GF) controls, displayed markedly elevated EBV infection rates and lymphoma incidence following oral viral challenge. Mechanistically, the microbiota depletes immune resources indirectly by expanding pools of viral target cells—specifically CCR5^+^ CD4^+^ T cells—thereby blunting CD8^+^ T cell-mediated viral surveillance (102). These data suggest that microbial context reshapes EBV-specific CD8^+^ T-cell competence by simultaneously altering both target-cell abundance and antigen-presenting cell quality.

Microbial dysbiosis during coinfection compromises EBV-specific CD8^+^ T cell competence via convergent mechanisms. Initially, coinfection drives immune checkpoint upregulation while blunting antigen presentation. EBV alone induces immunosuppressive ligands like PD-L1 on infected cells; coinfection amplifies this expression, fostering a tolerogenic niche. Mechanistically, EBV viral proteins such as LMP1 engage the STAT3/NF-κB axis (103), while viral miRNAs modulate PD-L1 transcription (81). Concurrently, coinfection-derived cytokines, including tumor necrosis factor-α (TNF-α) and IFN-γ, trigger JAK-STAT signaling (103). STAT3-associated signaling contributes to PD-L1 upregulation in EBV-associated tumors (104). Furthermore, coinfection erodes mucosal barriers—compromising intestinal or nasopharyngeal epithelial integrity. Such breach facilitates microbial translocation, causing chronic Toll-like receptor stimulation and dendritic cell (DC) tolerance. Consequently, DCs fail to efficiently present EBV antigens (105). Secondly, coinfection-associated gut dysbiosis further destabilizes mucosal integrity. This permits microbe-associated molecular patterns (MAMPs) to translocate into lymphoid compartments (102). MAMPs such as LPS engage TLR4/9, activating NF-κB signaling. This upregulates the EBV latent membrane protein LMP1 (106), forcing the virus from latency into lytic replication. The result is an elevated viral load and increased antigenic burden. Clinical data corroborate this: in HIV-EBV co-infected cohorts, intestinal *Bacteroides* enrichment correlates positively with plasma EBV-DNA loads. This link involves TLR9-mediated demethylation of the BZLF1 promoter—the master switch for EBV lytic reactivation (102). Such multilayered disruption converts coinfection from a coincidental accompaniment into an active driver of EBV immune escape.

#### 3.2.2 Microbial metabolites and taxa suppress NK cell effector function

Microbes and their metabolites curtail natural killer (NK) cell activity through diverse axes: bile acid dyshomeostasis, inflammatory cascade activation, epigenetic remodeling, and developmental imprinting. Gut microbiota critically governs NK cell homeostasis. Specific microbiota-derived short-chain fatty acids (SCFAs), notably butyrate, exert potent immunosuppressive pressures. Murine studies by Ma’s group reveal that early-life antibiotic exposure induces adult gut dysbiosis and depletes butyrate pools. This deficit impairs the maturation and antitumor efficacy of liver-resident NK (LrNK) cells. The mechanism hinges on reduced hepatocyte and Kupffer cell IL-18 secretion, which stalls LrNK functional maturation. Restoring butyrate-producing taxa reverses this suppression (107). In human peripheral blood NK cells, butyrate displays robust anti-inflammatory traits. Upon IL-12/IL-15 stimulation, butyrate exposure downregulates activating receptors TRAIL, NKp30, and NKp44. It also suppresses key effector molecules: IFN-γ, TNF-α, granzyme B, and perforin. Mechanistically, butyrate rewires NK metabolic programming by inhibiting mTORC1 activity and c-Myc expression, thereby constraining cytotoxic potential (108). Beyond metabolism, butyrate reshapes NK function epigenetically. As an HDAC inhibitor, it markedly elevates histone acetylation within NK cells, relaxing chromatin architecture (109). L-serine availability may also regulate NK-cell activity. In melanoma models, *Eubacterium* rectale depleted microenvironmental L-serine, which activated FOS/FOSL2 signaling and enhanced NK-cell-mediated antitumor immunity (110). Conversely, *Bacteroides ovatus* drives the production of iso-lithocholic acid (iso-LCA). When the key bile acid synthase AKR1D1 (aldo-keto reductase family 1 member D1) is deficient, *B. ovatus* expands, catalyzing the conversion of primary bile acid chenodeoxycholic acid (CDCA) into iso-LCA. This secondary bile acid suppresses CREB1 phosphorylation, blocking the secretion of interferon-γ (IFN-γ), tumor necrosis factor-α (TNF-α), and granzyme B in NK cells, which blunts their tumoricidal capacity. In hepatocellular carcinoma contexts, iso-LCA accumulation correlates strongly with diminished tumor-infiltrating NK cell counts and adverse patient prognosis (111). This underscores that microbiota-derived metabolites can either sustain or blunt NK-cell surveillance depending on the biochemical context they impose.

Direct microbial modulation of NK activity is equally critical. Within the hepatocellular carcinoma (HCC) niche, *Brevibacillus parabrevis* triggers acetylation of the retinoic acid-related orphan receptor γ (RORγ). This upregulates the E3 ubiquitin ligase NEDD4L, promoting ubiquitination and degradation of iron transporters (SLC39A14, SLC39A8, and STEAP3). The resultant drop in intracellular ferrous iron and reactive oxygen species (ROS) inhibits NK cell ferroptosis (112). Aberrant lipid metabolism, such as oleic acid buildup, induces epigenetic dysregulation via the c-Myc/P300/H3K27ac axis. This synergizes with ferroptosis blockade to impair function (113). While the former sustains a “hot phenotype” enhancing antitumor activity, microbial dysbiosis in most solid tumors predominantly suppresses NK function. As noted, early antibiotic exposure inflicts permanent damage on LrNK cells. These non-circulating, tissue-resident subsets rely on microbiota-derived signals such as SCFAs during a critical developmental window. Early-life microbial deprivation arrests LrNK precursor differentiation, manifesting as reduced mature LrNK numbers and compromised cytotoxicity (107). Thus, microbial control of NK biology extends from acute signaling events to developmental imprinting of tissue-resident cytotoxic populations.

Within the TME, microbial metabolites and tumor-derived factors may jointly impair NK-cell metabolism and function, fostering an immunosuppressive niche. Wei Haiming et al. demonstrated in lung cancer models that TGF-β enrichment in advanced TME drives aberrant fructose-1,6-bisphosphatase (FBP1) expression in NK cells. By inhibiting glycolysis, FBP1 reprograms NK metabolism, severely compromising survival and effector function. Pharmacological inhibition of FBP1 restores glycolytic flux and antitumor potency (114). Similarly, the malignant ascites microenvironment (ascTME) in ovarian cancer disrupts glucose metabolism and mitochondrial integrity in tumor-infiltrating NK cells. This precipitates lipid peroxidation and oxidative damage, culminating in cellular dysfunction. Treatment with the Nrf2 activator RTA-408 mitigates oxidative stress and partially rescues NK cytotoxicity (115). Evidence further suggests that fasting remodels NK metabolism, boosting fatty acid oxidation capacity to counteract TME-induced lipid stress (116). These findings suggest that metabolic dysfunction is one mechanism through which the tumor microenvironment can impair NK-cell activity.

The clinical weight of microbiota-NK cell crosstalk rests on its viability as a novel immunotherapeutic axis. Spironolactone, a structural isoform of iso-LCA, competitively antagonizes iso-LCA-mediated CREB1 suppression, thereby rescuing NK cell cytokine output. In murine tumor models, spironolactone co-administered with PD-1 blockade yielded synergistic antitumor potency, validating the strategy of targeting microbial metabolic nodes to potentiate NK surveillance (111). Yet, microbial metabolite activity remains strictly context-dependent. N-acetylglucosamine (GlcNAc) produced by the gut microbiome has been shown to modulate NK-cell responses and enhance influenza resistance (117). Conversely, within the tumor milieu, analogous mechanisms may be counteracted by immunosuppressive pressures.

### 3.3 Microbiome-associated remodeling of the immunosuppressive niche

EBV-driven oncogenesis is closely linked to evasion of host immune surveillance. The evidence reviewed here suggests that local and gut microbiota may contribute to this process by reshaping immune checkpoint activity, Treg/Th17 balance, and cytotoxic lymphocyte function. This ranges from upregulating pivotal checkpoints PD-L1 and CTLA-4 within the tumor stroma (76). Microbiota-derived SCFAs can modulate the Treg/Th17 balance (90). Microbiota-dependent bile acid metabolism can also modulate NK-cell cytotoxicity (111). These findings support the view that microbial factors may act not only as viral reactivation cues, but also as context-dependent regulators that cooperate with viral oncoproteins to weaken host immune clearance (2). The immunosuppressive niche is therefore best understood as a co-constructed product of viral persistence and microbial ecological pressure.

Importantly, this immune remodeling provides a direct conceptual bridge to the genomic instability discussed below. Under effective immune surveillance, EBV-infected cells carrying substantial DNA damage would be expected to undergo immune-mediated elimination by cytotoxic CD8^+^ T cells and NK cells. EBV-associated PD-L1 upregulation may contribute to immune evasion and thereby reduce immune-mediated clearance (118). Treg enrichment and T-cell exhaustion may further weaken immune surveillance, potentially allowing genetically damaged cells to persist and undergo clonal expansion (103).

However, direct evidence that specific microbial taxa or metabolites causally remodel EBV-specific antitumor immunity remains limited. Studies in melanoma, colorectal cancer, and gastric cancer have provided strong evidence that gut microbiota composition and microbial metabolites can regulate immune checkpoint activity, T-cell exhaustion, and responses to immune checkpoint blockade. In EBV-associated malignancies, evidence for PD-L1 upregulation, Treg enrichment, and impaired cytotoxic immunity is also substantial. However, fewer studies have directly tested whether specific microbial taxa or metabolites causally reshape EBV-specific CD8^+^ T-cell or NK-cell surveillance within EBV-positive tumors. Therefore, the immune-escape model proposed here should be viewed as a mechanistically coherent synthesis of EBV immunobiology and microbiome-immunotherapy research, rather than as a clinically validated causal pathway in all EBV-associated cancer types.

## 4 Inflammation and genomic instability

**Figure 5:**
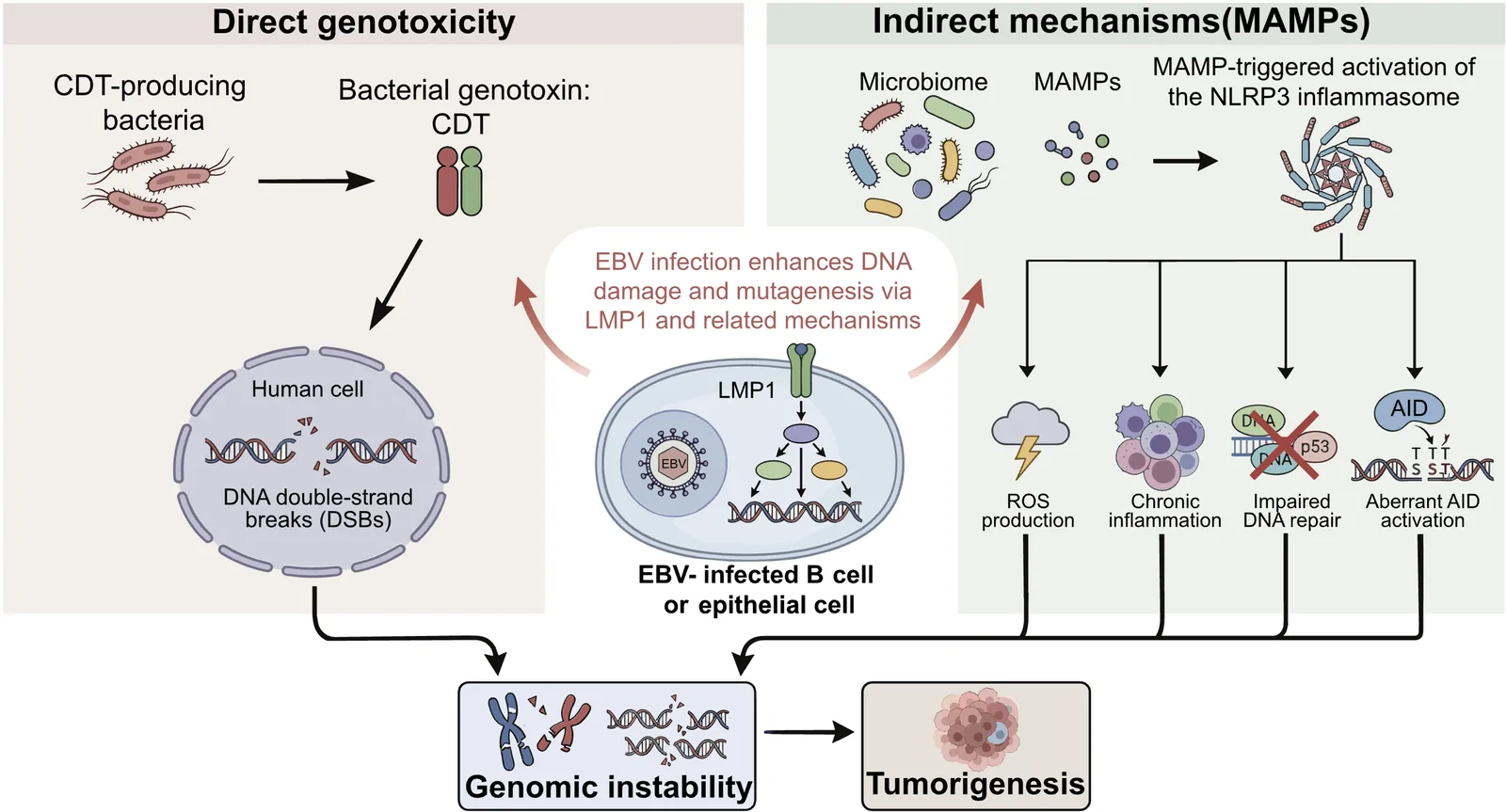
Microbiome–EBV cooperation in driving genomic instability. The left side illustrates direct genotoxic effects mediated by CDT-producing bacteria, including Aggregatibacter actinomycetemcomitans, whose cytolethal distending toxin (CDT) can induce DNA double-strand breaks and activate DNA-damage responses in host cells. The right side illustrates indirect mechanisms driven by microbiome-derived molecules, including microbe-associated molecular patterns (MAMPs) and metabolites, that activate innate immune pathways such as the NLRP3 inflammasome, promoting reactive oxygen species (ROS) production, chronic inflammation, impaired DNA repair, and aberrant activation-induced cytidine deaminase (AID) activity. In both pathways, EBVinfected cells expressing viral proteins such as LMP1 may enhance inflammatory and DNA-damage signaling. These interactions may promote genomic instability, mutation accumulation, and tumorigenesis.

Genomic instability represents a convergent endpoint of carcinogenesis. Epstein-Barr virus (EBV) proteins, notably EBNA1, can compromise DNA repair fidelity (58). Current evidence suggests that the microbiome may further exacerbate this genotoxic burden (**Figure 5**). Microbial factors and EBV may cooperate to promote genomic instability through persistent oxidative stress, direct genotoxin exposure, and epigenetic dysregulation (24). These findings suggest that microorganisms may act as context-dependent contributors to genotoxic stress whose effects complement those of viral oncoproteins. However, the extent to which these processes operate together in human EBV-associated tumors remains to be established.

Genomic instability is considered through two non-mutually exclusive routes: EBV reactivation-associated injury and reactivation-independent microbial mutagenesis. H. pylori infection can induce aberrant AID expression in gastric epithelial cells, providing one reactivation-independent route to genomic injury (22). Plasmodium infection can likewise promote AID-dependent genomic instability in B cells (23). Direct DNA damage caused by bacterial genotoxins such as cytolethal distending toxin represents another reactivation-independent route (119). EBV-associated disruption of DNA-damage responses may increase the persistence of microbially induced DNA lesions (120). Impaired antiviral immunity may further allow damaged cells to escape immune clearance (103).

### 4.1 Chronic inflammation as a precancerous precursor state

#### 4.1.1 Synergistic activation of NF-κB/STAT3 by microbiota and EBV LMP1

Gut microbiota can activate inflammatory NF-κB and STAT3 signaling through pattern-recognition pathways triggered by microbial metabolites and pathogen-associated molecular patterns such as lipopolysaccharide and flagellin. Rather than repeating the upstream TLR signaling cascade described above, this section focuses on how sustained NF-κB/STAT3 activation provides a chronic inflammatory platform for EBV-associated genomic damage. Sustained NF-κB signaling can promote pro-inflammatory cytokine production within the tumor microenvironment (121). STAT3 signaling can contribute to the maintenance of immunosuppressive cell populations within the tumor microenvironment (122). These changes may create a tissue environment in which DNA-damaged cells are more likely to persist. Accordingly, microbiota-driven NF-κB/STAT3 signaling should be interpreted as a shared inflammatory conduit linking dysbiosis, EBV signaling, and genomic instability.

EBV latent membrane protein 1 (LMP1) functions as a constitutively active mimic of tumor necrosis factor receptors. Via its C-terminal activation region, it recruits tumor necrosis factor receptor-associated factor complexes to activate NF-κB signaling (123). LMP1 activates NF-κB signaling by promoting IκBα degradation and p65 nuclear translocation (123). Experimental data confirm that LMP1 expression induces IκBα degradation coupled with p65 nuclear entry within 15 minutes; notably, a dominant-negative IκBα mutant completely abrogates this cascade (124). Through these unique assembly and activation mechanisms, LMP1 increases NF-κB transcriptional activity. The result is an upregulation of anti-apoptotic factors such as Bcl-2 and cell cycle regulators such as Cyclin D1, fostering proliferation while suppressing apoptosis (123). Concurrently, LMP1 reinforces immune evasion by activating STAT3: phosphorylated STAT3 drives programmed death-ligand 1 (PD-L1) expression, thereby inhibiting T cell function. In nasopharyngeal carcinoma models, tumor supernatant elevates PD-L1 on vascular endothelial cells via NF-κB/STAT3 crosstalk, significantly impairing T cell cytotoxicity; animal studies have validated that this mechanism accelerates tumor progression (80). In this setting, LMP1 does not merely mimic host receptor signaling, but may sustain inflammatory and prosurvival signaling.

The synergy between the microbiota and LMP1 profoundly exacerbates genomic instability. Sustained inflammatory signaling can increase ROS production through NADPH oxidases, thereby promoting oxidative DNA damage (125). Chronic oxidative stress may also contribute to telomere attrition (126). Inflammatory signaling may also contribute to genomic instability through APOBEC3B. In hepatocellular carcinoma cells, IL-6/JAK1/STAT3 signaling has been shown to increase APOBEC3B expression (127). APOBEC3B, in turn, can promote cytosine deamination and APOBEC-associated mutagenesis (128). In intestinal stem cells with downregulated SETDB1 expression, failed epigenetic silencing activates endogenous retroviruses. These produce ZBP1 ligand RNA, triggering RIP3–MLKL-mediated necroptosis and releasing damage-associated molecular patterns to establish a vicious cycle of chronic inflammation (129). Furthermore, *in vitro* LMP1 expression in epithelial cell lines inhibits DNA repair, inducing micronucleus formation, chromosomal aberrations, and genomic instability (130). Notably, patients with aggressive periodontitis show a significantly increased co-detection rate of EBV and periodontal pathogens including *Porphyromonas gingivalis* and *Aggregatibacter actinomycetemcomitans*, with EBV viral load positively correlating with disease severity (131). This microbe–EBV axis synergistically activates NF-κB/STAT3 signaling. The pathway fuels chronic inflammation and genomic instability while promoting epithelial–mesenchymal transition and stem-like traits, thereby driving malignant transformation.

#### 4.1.2 NLRP3 inflammasome activation drives tumor microenvironment remodeling

The NLRP3 inflammasome is an innate immune complex that may link microbial inflammation to tumor-associated tissue remodeling. Its activation follows a canonical two-step paradigm. First, microbe-associated molecular patterns (MAMPs) or damage-associated molecular patterns (DAMPs) engage Toll-like receptors or cytokine receptors such as TNFR and IL-1R, delivering the priming signal that upregulates NLRP3 and pro-IL-1β/IL-18 precursors. Subsequently, distinct danger signals—such as K^+^ efflux, ROS accumulation, or mitochondrial DNA release—trigger inflammasome assembly. This activates caspase-1, which processes pro-IL-1β and pro-IL-18 into their mature forms and promotes pyroptotic inflammatory responses (132). Microbial products such as LPS can provide inflammasome-priming signals through pattern-recognition receptor signaling (132). Within the tumor microenvironment (TME), this axis exerts dual regulatory control. Sustained NLRP3 signaling can promote tumor-associated inflammation and microenvironmental remodeling (133). Conversely, under specific contexts like post-chemotherapy, activation can bolster antitumor immunity. For instance, myeloid PTEN dephosphorylates NLRP3 at Tyr32, promoting IL-1β secretion. This significantly enhances CD8^+^ T cell infiltration and IFN-γ production, ultimately sensitizing tumors to chemotherapy (134). This duality indicates that the oncogenic significance of NLRP3 lies less in its mere activation than in the chronicity and tissue context of its activation.

Viruses, however, have evolved sophisticated mechanisms to subvert or aberrantly stabilize the NLRP3 inflammasome, evading immune clearance. The EBV-encoded G protein-coupled receptor (GPCR) BILF1 directly interacts with host MAVS. It recruits the ubiquitin-fold modifier 1 (UFM1) E3 ligase UFM1-specific ligase 1 (UFL1), inducing mitochondrial antiviral-signaling protein (MAVS) UFMylation. Modified MAVS is then packaged into mitochondria-derived vesicles for lysosomal degradation, effectively blocking the MAVS-mediated NLRP3 activation pathway (135). This strategy allows EBV to bypass NLRP3-triggered antiviral responses, facilitating persistent latent infection and subsequent oncogenesis. Furthermore, viral infections such as SARS-CoV-2 or type I interferons can induce ISG15 and E3 ISG ligases, including human HERC5 and murine HERC6. These enzymes catalyze NLRP3 ISGylation. This post-translational modification inhibits K48-linked polyubiquitination and proteasomal degradation, thereby stabilizing NLRP3 protein levels and potentiating inflammasome activity (136). In the context of persistent infection within the TME, such virus-mediated NLRP3 stabilization may perpetuate a chronic inflammatory state, driving genomic instability and carcinogenesis.

NLRP3 activation triggers pro-inflammatory cytokine release and pyroptosis. Pyroptosis releases damage-associated molecular patterns, which can sustain inflammatory signaling in the TME. This persistent state drives genomic instability. ROS and RNS accumulate at inflammatory foci, directly oxidizing DNA to induce point mutations and strand breaks (137). NLRP3 signaling also compromises the fidelity of core repair machineries—mismatch repair and base excision repair—blunting cellular repair capacity and accelerating mutational load (138). Such chronic inflammatory pressure imposes potent selective forces, favoring the clonal expansion of oncogene-bearing variants. Moreover, DAMPs liberated during pyroptosis amplify inflammatory signaling, establishing an “inflammation–damage–repair–proliferation” positive feedback loop. This cycle furnishes continuous mitogenic stimuli and an environment that may favor mutation accumulation and clonal selection, fueling malignant clone expansion (139). Persistent inflammasome signaling therefore transforms inflammatory injury from a transient defense response into a cumulative mutational engine.

NLRP3 inflammasome engagement remodels the TME across multiple dimensions, markedly boosting its tumor-promoting potential. First, NLRP3 drives IL-1β secretion, a pivotal upstream mediator of inflammation-associated angiogenesis that vigorously stimulates neovascularization to supply essential nutrients and oxygen (140). Second, NLRP3 activation and its associated cytokine milieu recruit immunosuppressive populations: MDSCs, tumor-associated macrophages (TAMs), particularly the M2 phenotype, and Tregs (141). These subsets secrete immunosuppressive mediators including IL-10, TGF-β, and VEGF and express inhibitory checkpoints such as PD-L1 and CTLA-4, collectively thereby suppressing antitumor immune responses (142). Finally, synergistic signaling by IL-1β and TGF-β potently drives EMT, enhancing tumor cell migration and invasion (143). Collectively, EBV–microbe axis-triggered NLRP3 activation reconfigures the local TME into an inflammation-rich niche conducive to tumor growth, immune evasion, and metastatic dissemination, thereby propelling carcinogenesis and progression.

### 4.2 Microbial drivers of mutagenesis

#### 4.2.1 Plasmodium-mediated AID activation propels MYC–IGH translocation in Burkitt lymphoma

Burkitt lymphoma stands as the paradigmatic aggressive B-cell malignancy. It is defined molecularly by the t (8, 14)(q24;q32) translocation in 75%–85% of cases. This rearrangement displaces the MYC proto-oncogene from chromosome 8 to the immunoglobulin heavy chain (IGH) locus on chromosome 14 (144). Sequestered under IGH enhancers, MYC undergoes constitutive overexpression, driving malignant transformation. In endemic zones like Africa, *Plasmodium falciparum* infection serves as a critical environmental cofactor (145). The underlying mechanism hinges on chronic antigenic stimulation. Persistent B-cell activation triggers aberrant expression and dysregulation of activation-induced cytidine deaminase (AID) (146). In this framework, *chronic Plasmodium* exposure supplies the mutational pressure that renders MYC translocation biologically and epidemiologically plausible.

AID functions as a potent oncogenic driver across diverse tumor types. Consider *Helicobacter pylori*: it, or its induced inflammatory mediators such as TNF-α, upregulates AID in gastric epithelium via NF-κB signaling (22). Similarly, in colitis, cytokines including TNF-α, IL-4, and IL-13 induce ectopic AID in colonic epithelial cells through NF-κB and STAT6 pathways, respectively (147). Such sustained AID expression in non-lymphoid compartments acts as a genomic mutagen. Deamination events introduce high-frequency point mutations or deletions in tumor suppressors like TP53, fueling carcinogenesis. This paradigm illuminates *Plasmodium*-driven lymphomagenesis. Physiologically, AID orchestrates somatic hypermutation and class switch recombination within Ig loci to diversify antibodies (23). Recurrent *P. falciparum* exposure can prolong B-cell activation and increase the opportunity for AID-mediated off-target lesions (23). Thus, *Plasmodium* infection emerges as a decisive environmental factor promoting AID-dependent genomic instability.

Epstein-Barr virus (EBV) serves as a pivotal cofactor in Burkitt lymphoma pathogenesis, establishing latent infection in >95% of endemic cases. These tumors predominantly exhibit Latency I, with viral gene expression largely restricted to EBNA1 and non-coding RNAs such as EBERs (1). While EBV does not directly catalyze the MYC–IGH translocation, it fuels genomic instability and drives malignant clone expansion through multifaceted mechanisms. LMP1-driven NF-κB and JAK/STAT signaling can promote a pro-inflammatory cytokine milieu, including IL-6 and IL-10 (148). Such an environment is critical for sustaining aberrantly high AID expression and enzymatic activity. EBV-associated metabolic remodeling can influence the epigenetic regulation of viral latency (51). Adequate methionine availability proves essential for maintaining the hypermethylated status of both the viral genome and the latency program. Metabolic conversion yields S-adenosylmethionine (SAM) from methionine, acting as the primary methyl donor. CpG methylation of EBV lytic-gene promoters contributes to the maintenance of viral latency (46). Furthermore, LMP1 can directly engage the AID promoter via Egr-1 transcription factor activation, indirectly amplifying NF-κB signaling (149). Mimicking CD40 engagement, LMP1 sustains PI3K/Akt and NF-κB pathway activation in B cells. This not only markedly upregulates AID levels but may also impede its degradation. Notably, EBNA2 remains absent in Latency I Burkitt lymphoma; its role likely confines to the early transformation phase, where it binds and activates the MYC promoter (150). EBV therefore acts as a molecular amplifier that stabilizes the inflammatory and epigenetic conditions required for AID-driven lymphomagenesis.

#### 4.2.2 Bacterial genotoxins trigger DNA damage in EBV-infected epithelial cells

Some mucosa-associated bacteria produce genotoxins that can directly damage host DNA. The cytolethal distending toxin (CDT), produced by Gram-negative species such as *Escherichia coli*, stands as the prototypical example (151). Its active subunit, CdtB, has DNase-like activity and can induce DNA double-strand breaks and activate host DNA-damage-response pathways (119). In EBV-infected epithelial cells, this injury correlates tightly with the viral latent-lytic switch. Experimental data indicate that CDT exposure triples viral reactivation rates in EBV-positive AGS gastric epithelial cells. This surge accompanies upregulated expression of lytic immediate-early genes BZLF1 and BRLF1, suggesting the toxin disrupts latency via DNA damage stress (24). Persistent genotoxin-induced DNA-damage responses can contribute to genomic instability (152). This synergy holds particular relevance in nasopharyngeal carcinoma (NPC) and EBV-associated gastric cancer (EBVaGC). Although *H. pylori* lacks CDT production, the oxidative stress and base lesions it induces potentiate DDR signaling in EBV-infected epithelia. Collectively, these mechanisms may increase genomic instability and facilitate malignant evolution (24). These observations support a model in which bacterial genotoxins may render EBV-infected epithelial cells particularly susceptible to mutation accumulation and malignant transformation.

The carcinogenic synergy between bacterial genotoxins and EBV orchestrates cell fate through multi-layered regulation. This interplay transcends simple additivity: bacterial genotoxins amplify the genomic fragility instigated by EBV, while the viral latency program fosters a permissive niche for mutagenized clones. By systematically subverting the DNA damage response (DDR), blocking apoptosis, and driving proliferation, EBV ensures the survival and clonal expansion of genotoxin-compromised cells. Initially, genotoxin-induced double-strand breaks (DSBs) trigger ATM/Chk2 signaling, arresting the cell cycle at G2/M (153). USP7 is an important regulator of EBNA1 stability, and pharmacological inhibition of USP7 can destabilize EBNA1 (154). In EBV-immortalized nasopharyngeal epithelium, this arrest often correlates with LMP1 expression. LMP1 attenuates DDR efficiency by suppressing γH2AX phosphorylation yet promotes survival via NF-κB signaling, enabling damaged cells to evade apoptotic clearance (120). Furthermore, chronic exposure to colibactin from *E. coli* B2 lineages induces senescence, marked by telomere dysfunction-induced foci and p16INK4a/p53 pathway activation (155). In EBV-positive B-cell models, such telomeric attrition drives a senescence-associated secretory phenotype (SASP). SASP-associated cytokine release may contribute to remodeling of the tumor microenvironment (156). Animal studies confirm that repeated CDT exposure accumulates oncogenic hits in genes such as APC and KRAS within intestinal epithelia (157). Concurrent EBV infection accelerates malignant transformation by expressing viral miRNAs, notably the BART cluster, which suppress critical DDR factors including ATM and p53 (158). The decisive outcome is therefore not DNA injury alone, but the survival and clonal expansion of cells that should otherwise have been eliminated.

Molecular epidemiology validates the clinical relevance of this “microbe–virus” axis. In EBV-positive gastric cancer, *H. pylori* coinfection is prevalent; these co-infected specimens exhibit elevated DNA oxidative damage and stress markers (159). Similarly, within the oropharyngeal microbiome of patients from NPC-endemic regions, the abundance of CDT-producing taxa such as *Aggregatibacter actinomycetemcomitans* positively correlates with EBV viral load (160). Mechanistically, bacterial toxins drive carcinogenesis not only via direct genotoxicity but also by modulating the immune landscape (161). CDT induces premature senescence in activated CD4^+^ T cells, triggering SASP and a surge in cytokine and chemokine expression. This dysregulation impairs broader immune function, eroding host anti-infective capacity and thereby sustaining persistent viral infection (162). Clinical co-detection data thus reinforce the view that microbial genotoxicity and viral persistence cooperate within the same pathogenic field rather than in parallel isolation.

### 4.3 Reactivation-independent microbial mutagenesis driving mutational load

Beyond reactivation-associated genomic injury, this section focuses on direct microbial mutagenesis, including AID-mediated lesions and bacterial genotoxin-induced DNA damage, and its potential cooperation with EBV-associated defects in genome maintenance.

A critical limitation of this evidence is that direct microbial mutagenesis and EBV-mediated genomic instability have often been studied in separate experimental contexts. Controlled experimental studies show that cytolethal distending toxin can induce DNA double-strand breaks and activate DNA-damage responses (24). Colibactin-producing E. coli can likewise induce DNA double-strand breaks in eukaryotic cells (163). In contrast, evidence that these genotoxic processes operate simultaneously with EBV latency programs in human tumors remains limited. Similarly, AID-mediated mutagenesis is well supported in malaria-associated lymphomagenesis and *H. pylori*-associated epithelial injury, but the extent to which AID acts as a generalizable mechanism across EBV-positive epithelial cancers remains uncertain. Therefore, microbial mutagenesis should be interpreted as a complementary mechanism that may cooperate with EBV-driven repair defects, rather than as a universally established driver across all EBV-associated malignancies.

## 5 Targeting the microbiome–EBV axis

**Figure 6:**
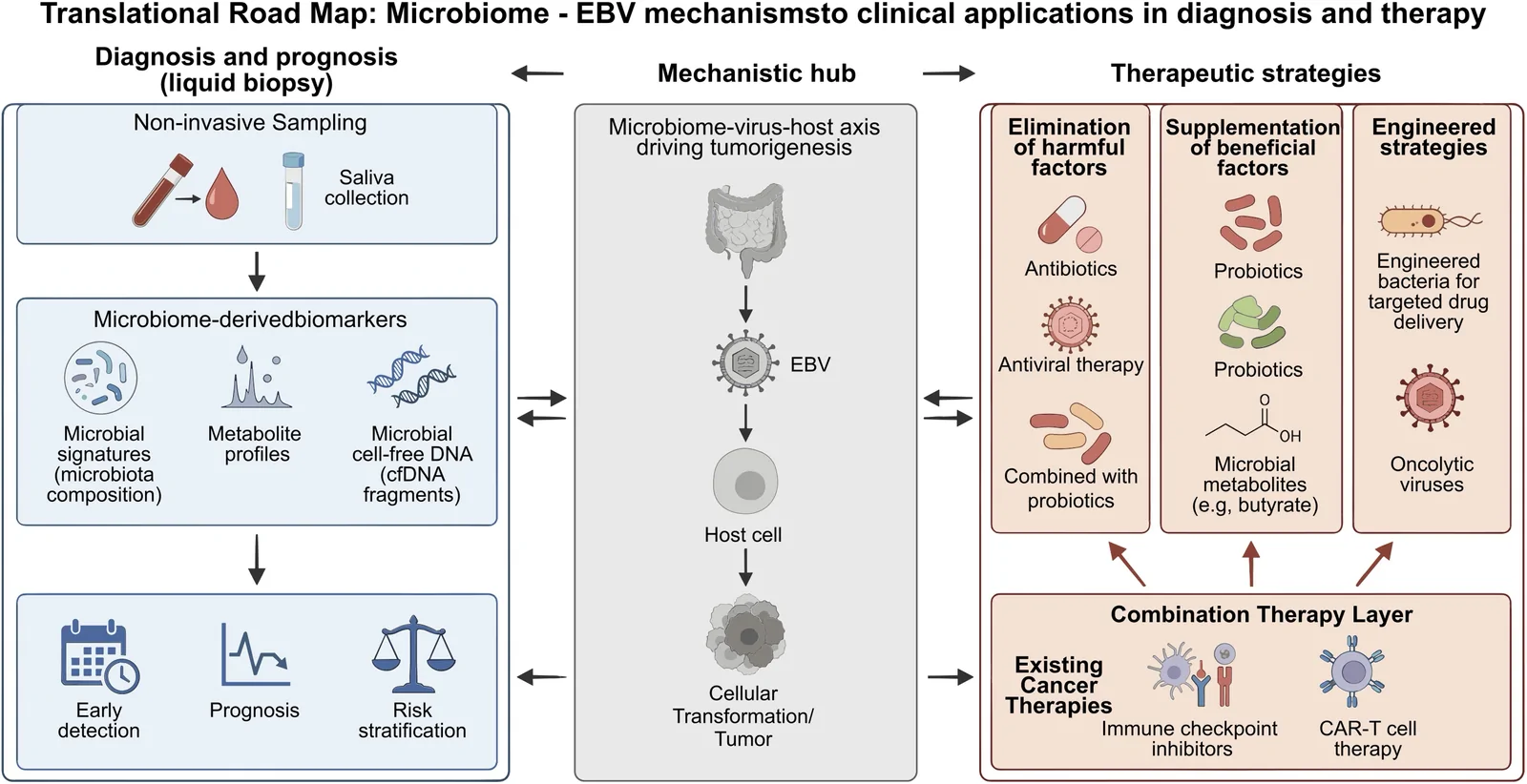
Translational roadmap of the microbiome–EBV axis for diagnosis and therapy. The mechanistic microbiome–virus–host hub is linked bidirectionally to clinical applications. On the diagnostic side, non-invasive blood and saliva sampling enables analysis of microbial signatures, metabolite profiles, and microbial cell-free DNA for early detection, prognosis, and risk stratification. On the therapeutic side, interventions include elimination of harmful factors using antibiotics, antivirals, and probiotic-supported regimens; supplementation with beneficial components such as probiotics, prebiotics, and microbial metabolites; and engineered approaches including targeted bacteria and oncolytic viruses. These strategies integrate with immune checkpoint blockade and CAR-T cell therapy to support precision treatment of EBV-associated malignancies.

The mechanistic framework of the microbiome–EBV axis may inform the development of translational strategies. This chapter examines how this framework may be applied to EBV-associated malignancies (**Figure 6**). If the microbiome contributes to EBV-associated carcinogenesis, microbiome modulation may provide an indirect means of reducing viral and tumor-promoting signals and modifying the tumor microenvironment. This approach adds ecological intervention to existing strategies based on direct cytotoxicity or viral suppression (2). Such interventions may reduce the development of precancerous lesions or improve the efficacy of existing treatments. Operationally, these strategies can be categorized according to the pathogenic steps they aim to modify: eliminating microbial triggers, restoring beneficial microbes or metabolites, reversing microbiome-associated immune suppression, and exploiting controlled EBV lytic induction for selective killing of EBV-positive cells. This classification links the mechanistic framework described above to the intervention strategies discussed below. The following sections discuss preventive and therapeutic approaches. Prebiotic or probiotic supplementation may support mucosal barrier function and reduce low-grade inflammation (164), potentially lowering the likelihood of EBV reactivation (28). Moreover, in specific high-risk ecologies—notably malaria-endemic zones linked to Burkitt’s lymphoma—the targeted deployment of antimalarials or antibiotics to eradicate key “co-pathogens” (165) emerges as a vital primary prevention tactic, severing the synergistic chain of microbial-EBV oncogenesis (166). The aim is to intervene before malignant transformation occurs.

### 5.1 From ecological distribution to a synergistic carcinogenic network

Before the microbiome–EBV axis can be translated into clinical practice, two questions require clarification: whether shared mechanisms operate across geographic regions and cancer types, and how distinct pathways interact over time. Current evidence suggests that shared signaling mechanisms coexist with tissue- and context-specific microbial factors.

#### 5.1.1 Universality and tissue specificity of the microbiome–EBV axis

The pathogenic axis linking the microbiome and EBV appears to operate through shared signaling conduits across diverse populations and malignancies characterized by distinct epidemiological signatures—ranging from nasopharyngeal carcinoma in South China and gastric cancer in East Asia to lymphoma in equatorial Africa. Yet, beneath this umbrella of commonality lie independent, tissue-specific molecular interaction patterns that dictate the unique trajectory of each disease.

From a translational perspective, the microbiome–EBV axis appears to converge on two recurrent mechanistic modules: an inflammatory module involving PAMP–TLR–NF-κB signaling and a metabolic–epigenetic module involving SCFAs and HDAC-dependent chromatin regulation. Microbial-trigger control is supported by evidence linking oral microbiota to EBV reactivation (32). Microbial metabolites can regulate host immune-cell differentiation (167). Microbial metabolites can also influence EBV chromatin regulation (52). Inflammatory and immune-pathway modulation may also be relevant to EBV-positive tumors.

Yet, at the level of ecological specificity, the manifestation of these axes is not monolithic; rather, it is sculpted by the unique confluence of local tissue niches and viral host cell tropism. The emergence of EBV-associated malignancies hinges critically on “Anchor Microbes” distinct to particular anatomical sites. In nasopharyngeal carcinoma, oral microbiome alterations have been associated with EBV reactivation (32). Bacterial genotoxins such as cytolethal distending toxin provide a separate preclinical mechanism linking microbial exposure to DNA damage and EBV reactivation (24). Conversely, in East Asian gastric cancer, the acid-resistant niche of the gastric mucosa renders *Helicobacter pylori* an indispensable synergistic carcinogen; its coinfection with EBV significantly exacerbates host DNA damage responses and genomic instability. In equatorial Africa, the dynamics shift entirely for endemic Burkitt’s lymphoma (eBL): here, systemic, recurrent *Plasmodium falciparum* infections supplant local mucosal flora as the primary environmental trigger, driving chronic B-cell activation, inducing aberrantly high expression of activation-induced cytidine deaminase (AID), and ultimately facilitating *MYC* gene translocation (23). Furthermore, the latent programs established by EBV vary fundamentally by target cell—epithelial cells versus B lymphocytes. The contrast between the highly restricted latency type I in Burkitt’s lymphoma and the LMP1-expressing latency type II in nasopharyngeal carcinoma (23) implies that identical microbial metabolites or inflammatory signals can precipitate downstream carcinogenic cascades with distinctly different emphases, contingent upon the host cell context.

#### 5.1.2 Cross-pathway interactions within the carcinogenic network

Crucially, within the actual tissue microenvironment, pathways defined by core commonalities and tissue specificity do not function as isolated, parallel linear conduits. Rather, through profound crosstalk, they interlace to form a dynamic, synergistic carcinogenic closed loop spanning “epigenetics-inflammation-mutation-immunity” (2). This systems-level mechanistic integration elegantly reconciles biological effects that appear contradictory when viewed through the lens of single pathways. Butyrate, acting as an HDAC inhibitor, can promote EBV lytic reactivation (52). Microbiota-derived metabolites such as butyrate can also promote regulatory T-cell generation (167). In traditional linear paradigms, viral lysis implies target cell apoptosis and immune exposure, an outcome seemingly at odds with carcinogenic logic. Yet, within the integrated carcinogenic network, this duality may represent a threshold-dependent interaction between lytic induction and immune suppression.

Regarding temporal dynamics and concentration thresholds, the chronic, gradient concentrations of butyrate and pro-inflammatory signals such as the LPS-activated TLR/NF-κB pathway prevalent in the tumor microenvironment are often insufficient to initiate a complete lytic cycle culminating in cell disintegration. Instead, incomplete or abortive lytic activation may occur under some conditions (1). In this state, cells exhibit limited expression of highly pro-transforming immediate-early or early viral proteins such as Zta and Rta, accompanied by a massive accumulation of reactive oxygen species (ROS), thereby contributing to the initiation of genomic instability (1). Subsequently, in the spatial dimension, these nascent transformed cells expressing aberrant antigens should theoretically face imminent clearance by CD8^+^ T cells. Here, however, secondary crosstalk emerges between microbial metabolites such as butyrate within the same microenvironment and tumor-derived factors such as TGF-β secreted by mutated cells. Butyrate and related microbial metabolites can promote Treg differentiation (167). PD-L1 upregulation can further contribute to immune escape in EBV-associated tumors (118). This temporal and spatial coupling helps explain how incomplete lytic activation can paradoxically facilitate, rather than abort, malignant progression.

In essence, the “microbiome–EBV axis” represents a complex system woven from universal signaling networks, specific ecological triggers, and dynamic cross-pathway closed loops. Future clinical translation should avoid a “one-size-fits-all” approach and instead combine shared immune or metabolic strategies with tissue-specific control of relevant co-pathogens. However, these combined strategies require disease-specific clinical evaluation.

### 5.2 Microbiome-targeted intervention strategies

#### 5.2.1 Potential probiotic modulation of inflammation and EBV reactivation

The interplay between gut microbial ecology and Epstein-Barr virus (EBV) dynamics represents a potential therapeutic target, because probiotics may modulate the latency–lytic switch by supporting barrier integrity and modulating immune responses. Specific Bifidobacterium strains may support intestinal epithelial barrier integrity (168). Some probiotic strains can also modulate innate and adaptive immune responses (169). Extracellular vesicles derived from *Bifidobacterium* (BEVs) can traverse the epithelial barrier and deliver immunomodulatory cargo to distant lymphoid sites. Through the TLR4-NF-κB axis, these BEVs drive dendritic cell maturation and CD8^+^ T cell infiltration while boosting interferon-γ (IFN-γ) secretion. The result is heightened antiviral surveillance that stifles lytic replication (30). Extracellular vesicles from specific lactic acid bacteria can exhibit agonistic activity toward FPR2 (170). Lactobacillus paracasei-derived extracellular vesicles can attenuate intestinal inflammatory responses (171). Such signaling shifts favor an M2 macrophage phenotype, attenuating the NF-κB-mediated inflammatory cascade (172), which is a primary driver of EBV immediate-early genes like *BZLF1*. Furthermore, oral *Bifidobacterium* appears to synergize with anti-programmed death-ligand 1 (PD-L1) therapy, likely by refining dendritic cell function to prime and accumulate CD8^+^ T cells (29). Beyond natural strains, engineered probiotics such as *Escherichia coli* Nissle 1917 (EcN) serve as delivery platforms. Equipped with radiation-responsive promoters, they release single-chain antibodies against TREM2 and reprogram tumor-associated macrophages from immunosuppressive M2 to pro-inflammatory M1 states in experimental tumor models (173). Collectively, these findings provide an indirect rationale for evaluating probiotic strategies in EBV-associated diseases.

Microbial metabolites may influence EBV-related pathways, although most available evidence remains indirect. Butyrate is a microbial HDAC inhibitor (174). Butyrate can also promote EBV lytic reactivation (175). Indole-3-lactic acid has shown barrier-protective effects in non-EBV experimental models (176). Propionate production has been associated with antitumor effects in a non-EBV colorectal cancer model (177). Their effects on the EBV latency–lytic switch remain unestablished. These metabolites should therefore be considered candidate mediators requiring direct validation in EBV-positive models.

Preclinical and early clinical findings from broader cancer immunotherapy and chronic viral disease contexts provide indirect support for exploring probiotics within the microbiome–EBV axis. In melanoma models, an oral Bifidobacterium-based vaccine triggered systemic antitumor immunity by activating CD8^+^ T cells and promoting central memory T-cell formation (178). These findings provide an immunological rationale for testing analogous approaches in EBV-associated malignancies, but they do not constitute direct evidence in EBV-driven tumor models. Similarly, probiotic-associated spermidine enhanced IFN-γ secretion and reduced serum hepatitis B surface antigen levels in patients with chronic hepatitis B. Because IFN-γ has also been implicated in regulating the EBV lytic cycle (179), this observation provides a hypothesis-generating rationale for future EBV-focused studies rather than direct clinical evidence for EBV-associated disease management.

Important research gaps remain. Most studies have focused on bacterial components, including extracellular vesicles and metabolites, leaving the direct impact of probiotics on EBV latency largely unexplored. Furthermore, existing evidence relies heavily on animal models or small-scale cohorts, with a notable absence of randomized controlled trials focused on EBV-related cancers. Future studies should use single-cell and multi-omics approaches to map the dynamic interplay between the microbiota, EBV, and the tumor microenvironment. Larger cohorts will be needed to develop more precise microbiota-based intervention strategies.

#### 5.2.2 Antibiotic and antimalarial interventions for high-risk populations

Antibiotic or antimalarial interventions may influence the microbiome–EBV axis in selected high-risk populations, such as immunocompromised individuals or residents in endemic zones. The aim is to modify microbial or coinfection-related factors that may contribute to EBV-associated oncogenic risk.

Antibiotics can substantially alter gut microbiome composition and function (180). Gut microbes and their metabolites contribute to immune maturation and homeostasis. For instance, the abundance of specific commensals such as *Akkermansia muciniphila* and *Bifidobacterium longum* correlates positively with immune checkpoint inhibitor efficacy (181), suggesting that microbiota composition may influence antitumor responses. Targeted antimicrobial treatment could theoretically reduce microbial taxa associated with inflammation or EBV reactivation, but direct evidence that this approach suppresses EBV lytic reactivation through microbiome modulation remains lacking. Broad-spectrum antibiotics also lack specificity and may deplete beneficial commensals, potentially compromising host immune surveillance (182). These limitations highlight the complexity and potential risks of antibiotic intervention. Future studies should prioritize narrow-spectrum antimicrobial strategies and relevant *in vivo* models to determine their effects on microbiome composition, EBV reactivation, and host immunity.

By comparison, antimalarial therapy represents a more established indirect approach to reducing EBV-associated risk, particularly within malaria-endemic zones. Repeated Plasmodium falciparum exposure is associated with increased EBV load in children living in malaria-endemic regions (183). Mechanistically, specific malaria antigens—notably CIDR1α—can directly engage latently infected B cells, promoting lytic reactivation and viremia (184). Compounding this, chronic malaria erodes EBV-specific T-cell immunity, further loosening the host’s grip on the virus (185). Antimalarial treatment has been associated with clearance of circulating EBV DNA in children with acute malaria (186). These findings suggest that controlling *P. falciparum* infection may reduce an external stimulus associated with EBV reactivation. Yet, while current efforts focus on quantifying the impact of antimalarials on viral load, the potential mediating role of gut or oropharyngeal microbiome modulation remains unresolved.

### 5.3 Adjuvant therapies

#### 5.3.1 Fecal microbiota transplantation for the restoration of immune homeostasis

Microbiome-modulating strategies may contribute to the restoration of host immune homeostasis. A case report described clinical improvement after washed microbiota transplantation (WMT) in a patient with acute severe ulcerative colitis (UC) complicated by viral myocarditis. The reported effects may be associated with changes in the intestinal immune environment (187). These findings suggest that fecal microbiota transplantation (FMT) may help restore immune homeostasis.

Other microbiome-based interventions are also under investigation. Engineered bacterial strains, such as attenuated Salmonella, have been investigated as delivery platforms for antineoplastic agents. Together, these findings support further evaluation of both whole-community transplantation and targeted microbial interventions for immune modulation. To optimize efficacy, safety, and applicability, current research is moving from conventional FMT protocols toward the rigorous identification and screening of specific microbial constituents. The objective is to isolate and harness the core functional taxa or molecules that contribute to therapeutic outcomes.

FMT is being investigated for its capacity to restructure gut microbial communities and influence host immune homeostasis and therapeutic response. Within the microbiome–EBV axis, FMT may improve systemic antitumor immunity by modulating immune dysfunction, whereas its direct effects on EBV-associated oncogenic processes remain uncertain. This possibility requires further investigation in EBV-associated malignancies. The underlying mechanism involves the engraftment of functional microbiota from healthy donors, which significantly augments the abundance of beneficial taxa such as *Bifidobacterium* and *Faecalibacterium*. These microbial changes have been associated with increased CD8^+^ T-cell infiltration and activation and reduced abundance of immunosuppressive populations, including IL-8-positive myeloid cells. Early-phase clinical studies in melanoma suggest that fecal microbiota transplantation combined with anti-PD-1 therapy may restore treatment responsiveness in a subset of patients (31). A recent meta-analysis further supports this therapeutic potential, although the available clinical evidence remains limited (188). These findings support further evaluation of FMT as a microbiome-based strategy for improving antitumor immunity.

Gut microbiota-derived metabolites, including short-chain fatty acids (SCFAs) such as butyrate, can link microbial activity to host epigenetic regulation (189). This mechanism may also be relevant to EBV-infected cells. During viral latency, low histone acetylation at the promoters of EBV lytic genes contributes to transcriptional repression (52). Butyrate exposure has been associated with increased histone acetylation at the BZLF1 promoter (52). As a key regulator of the EBV lytic cycle, BZLF1 undergoes transcriptional derepression through this epigenetic modification, thereby triggering the transition from latency to lytic replication (190). One hypothesis is that FMT may introduce SCFA-producing microbiota and alter systemic metabolite profiles. These metabolites subsequently act upon EBV-infected tumor cells to induce viral reactivation.

Notwithstanding the potential of FMT to modulate immune homeostasis and influence EBV-driven oncogenesis, its clinical safety warrants rigorous scrutiny, particularly in cancer patients with compromised immunity. A key question is whether FMT affects the risk of EBV reactivation during immunosuppression. In recipients of allogeneic hematopoietic stem cell transplantation (allo-HSCT), EBV reactivation represents a severe complication closely correlated with the depth of immunosuppression (191). Although prospective cohort data detailing EBV viral load dynamics post-FMT remain scarce (192), standard donor screening protocols typically incorporate serological testing for pathogens like EBV to mitigate transmission risks (193). Nevertheless, rare case reports indicate that EBV-related complications may arise even when donors are screened as seronegative (193). Therefore, clinical evaluation of FMT in EBV-associated malignancies would require appropriate risk stratification and longitudinal monitoring of EBV load.

#### 5.3.2 Microbial metabolites as modulators of EBV gene expression

Microbial metabolites are being investigated as potential regulators of the microbiome–EBV axis. One potential strategy is to use microbial metabolites to regulate EBV oncoprotein expression. Potential targets include Latent Membrane Protein 1 (LMP1) and Epstein-Barr Virus Nuclear Antigen 2 (EBNA2). EBNA2 is a key determinant of EBV-mediated B-cell transformation (150). LMP1 contributes to EBV-associated oncogenic signaling and cellular transformation (194). LMP1 constitutively activates host signaling pathways such as NF-κB, MAPK, and JAK/STAT. This activation promotes cell proliferation and survival and contributes to resistance to apoptosis (194). EBNA2 functions as a viral transcriptional regulator and is required for efficient EBV-mediated B-cell transformation (150). Identifying metabolites that regulate these oncoproteins may provide new therapeutic approaches for EBV-associated malignancies.

Short-chain fatty acids (SCFAs), especially butyrate, have been widely studied. This interest reflects their established epigenetic regulatory functions. Butyrate is a well-characterized histone deacetylase (HDAC) inhibitor (174). HDAC inhibition increases histone acetylation and can alter chromatin accessibility and transcriptional activity (195). This epigenetic effect provides a rationale for investigating whether butyrate modulates EBV oncoprotein expression.

However, because butyrate acts broadly, its effects on gene expression are complex and context dependent. For example, butyrate has been associated with epigenetic regulation of cell-cycle-related genes, including p21 (196). It may also inhibit proangiogenic factors and reduce tumor angiogenesis (195). These context-dependent effects complicate predictions regarding specific EBV oncoprotein promoters. Current evidence supports butyrate-mediated changes in gene expression through histone acetylation marks like H3K27ac (197). However, direct evidence that butyrate inhibits LMP1 or EBNA2 through this mechanism remains lacking. Similarly, other short-chain fatty acids lack direct evidence for suppressing LMP1 and EBNA2 expression. Therefore, whether microbial metabolites can directly inhibit EBV latent oncoproteins remains unclear.

Although direct suppression of latent oncoproteins remains unconfirmed, butyrate can promote the transition from EBV latency to the lytic cycle (25). As an HDAC inhibitor, butyrate can activate EBV lytic genes such as BZLF1. This activation can initiate viral lytic replication. This process may enhance the efficacy of concurrent antiviral therapy. Studies have shown that butyrate increases viral thymidine kinase (TK) expression (190). This enzyme facilitates the conversion of the antiviral prodrug Ganciclovir (GCV) into its cytotoxic triphosphate form. This process can promote the selective killing of EBV-positive malignant cells. Clinical studies have evaluated arginine butyrate combined with ganciclovir in EBV-positive lymphoma (198). Silencing key oncoproteins like LMP1 and EBNA2 via microbial metabolites remains hypothetical. However, modulation of the EBV latency–lytic switch has translational potential. Future studies should use chromatin immunoprecipitation sequencing and quantitative proteomics to determine how microbial metabolites regulate the EBV life cycle. Despite these opportunities, microbiome-targeted interventions for EBV-associated cancers require careful evaluation of specificity, safety, and stability. Broad-spectrum antibiotics may reduce microbial triggers but can also deplete beneficial commensals and impair host immune surveillance (199). Butyrate exerts immunomodulatory effects on host cells (164). Butyrate can also induce EBV lytic reactivation through mechanisms associated with HDAC inhibition and chromatin remodeling (200). Live biotherapeutic interventions require particular caution in immunocompromised patients because of potential infectious and safety risks (105). Therefore, future clinical translation should prioritize defined microbial consortia, targeted metabolite delivery, standardized preclinical models, and carefully stratified patient populations.

### 5.4 Translational relevance: biomarkers, microbiome modulation, and clinical evidence

The translational relevance of microbiome research in EBV-associated diseases can be considered at three levels: diagnostic biomarker discovery, therapeutic intervention, and clinical validation. At the diagnostic level, microbial signatures, microbial metabolites, and microbial cell-free DNA may provide noninvasive or minimally invasive tools for risk stratification, early detection, prognosis, and treatment-response prediction. Oral microbial alterations have been associated with EBV reactivation in nasopharyngeal carcinoma (32). Intratumoral microbiota have been associated with prognosis in nasopharyngeal carcinoma (201). Gastric microbial profiles have been associated with EBV status in gastric cancer (74). However, most current biomarker evidence remains observational, and candidate microbial markers require validation in multicenter prospective cohorts before clinical implementation.

At the therapeutic level, microbiome modulation may complement existing EBV-directed strategies through several routes. These include eliminating pathogenic microbial triggers, restoring beneficial commensals, supplementing or inhibiting microbial metabolites, and reshaping the tumor immune microenvironment. Among these strategies, the most clinically developed EBV-related example is lytic-induction therapy combined with antiviral treatment, as illustrated by arginine butyrate plus ganciclovir in EBV-positive lymphoid malignancies (198). By contrast, most microbiome-targeted interventions remain preclinical or early translational in EBV-associated cancers, although FMT-based microbiome modulation has shown clinical activity in broader cancer immunotherapy settings (202).

Ongoing clinical studies further illustrate both the promise and limitations of this field. A phase II randomized trial is evaluating fecal microbiome transplantation among recipients of CAR-T therapy (203). Another phase II trial is evaluating EBV lytic reactivation therapy combined with PD-1 blockade in recurrent or metastatic nasopharyngeal carcinoma (204). Nevertheless, direct clinical validation of microbiome-targeted interventions in EBV-associated cancers remains limited.

## 6 Future research directions

Future research on the microbiome–EBV axis should move from associative observations toward causal and clinically actionable evidence. Although microbial dysbiosis has been described in several EBV-associated cancers, most existing studies remain cross-sectional and cannot establish whether microbial changes are causal or secondary to disease-associated ecological shifts. Therefore, multicenter longitudinal cohorts, paired profiling of microbiota and EBV status, and controlled experimental models are needed to define causal relationships among microbial communities, EBV latency or reactivation, immune remodeling, and tumor progression (102). This distinction is essential because associative microbial signatures may have diagnostic or prognostic value even when they are not proven causal drivers of EBV-associated carcinogenesis.

Another important limitation is the heterogeneity and occasional inconsistency of the existing literature. Microbial signatures reported in nasopharyngeal and gastric cancer cohorts are not directly interchangeable across tissues or populations. Differences in sampling site, geography, treatment exposure, diet, antibiotic use, and analytical methods should therefore be considered when comparing studies. For example, microbial alterations identified in nasopharyngeal carcinoma cohorts from endemic regions may not be directly generalizable to gastric cancer, lymphoma, or non-endemic populations. Methodological variability may also contribute to inconsistent findings, including differences in sample collection, DNA extraction, sequencing approaches, taxonomic annotation, contamination control, and statistical adjustment for confounders. Therefore, conflicting microbial signatures should not necessarily be interpreted as mutually exclusive; rather, they may reflect context-dependent microbiome–EBV interactions shaped by host, tissue, geography, and methodology. Future studies should adopt standardized sampling and sequencing protocols, report key clinical and environmental covariates, and validate candidate microbial biomarkers across independent multicenter cohorts.

Multi-omics integration should be used to answer these unresolved biological and clinical questions rather than serving as a purely methodological expansion. Single-cell profiling can resolve EBV-associated immune states and treatment-related changes at high cellular resolution (67). Metagenomic and machine-learning approaches may support the identification of microbiome-based response biomarkers, although EBV-specific validation remains necessary (205). AI-assisted biomarker discovery may further support patient stratification by integrating microbial, viral, immune, and clinical variables into predictive models, although such models require EBV-specific validation.

**Figure 7:**
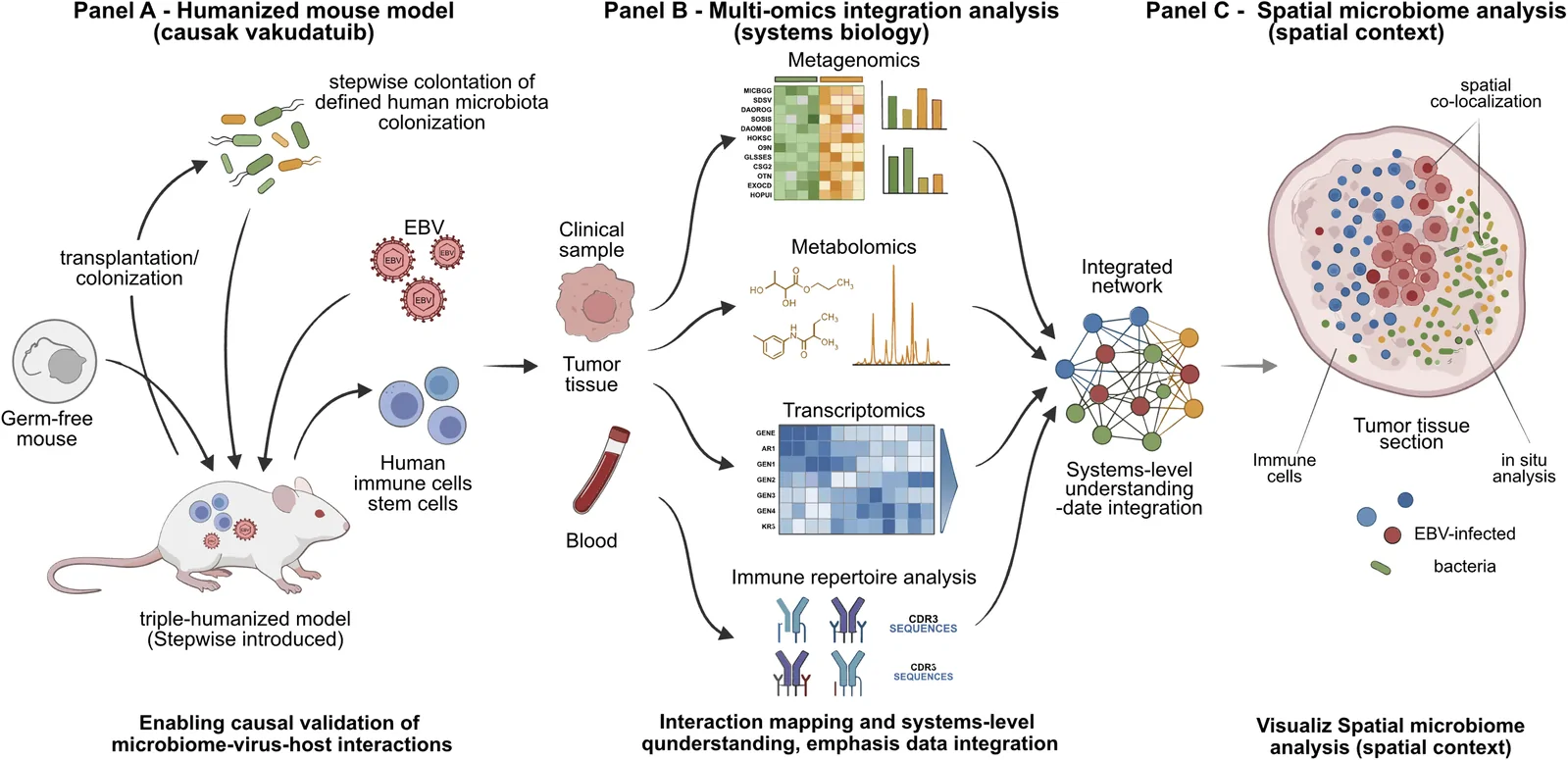
Advanced models and analytical frameworks for studying the microbiome–EBV axis. **(A)** A triple-humanized germ-free mouse model integrating human immune reconstitution, defined microbiota colonization, and EBV infection for causal validation of microbiome–virus–host interactions. **(B)** Multi-omics analysis of clinical samples integrating metagenomics, metabolomics, transcriptomics, and immune repertoire profiling into a unified interaction network. **(C)** Spatial analysis of tumor tissues showing the *in situ* co-localization of microbiota, EBV-infected tumor cells, and immune populations. Together, these approaches provide complementary platforms for causal inference, systems-level mapping, and spatially resolved investigation of microbiome–EBV-driven tumorigenesis.

Another priority is the development of more precise microbiome-engineering and personalized therapeutic strategies. Future interventions should move beyond broad-spectrum antibiotics, empirical probiotics, or conventional fecal microbiota transplantation toward defined microbial consortia, engineered probiotics, targeted metabolite modulation, and rational combinations with antiviral therapy, immune checkpoint blockade, or EBV-directed cellular therapy. Overall, the key challenge is to translate the microbiome–EBV framework into clinically testable approaches for risk prediction, prevention, and treatment. Advanced models and analytical frameworks for studying the microbiome–EBV axis are summarized in **Figure 7**.

## 7 Conclusions

**Figure 8:**
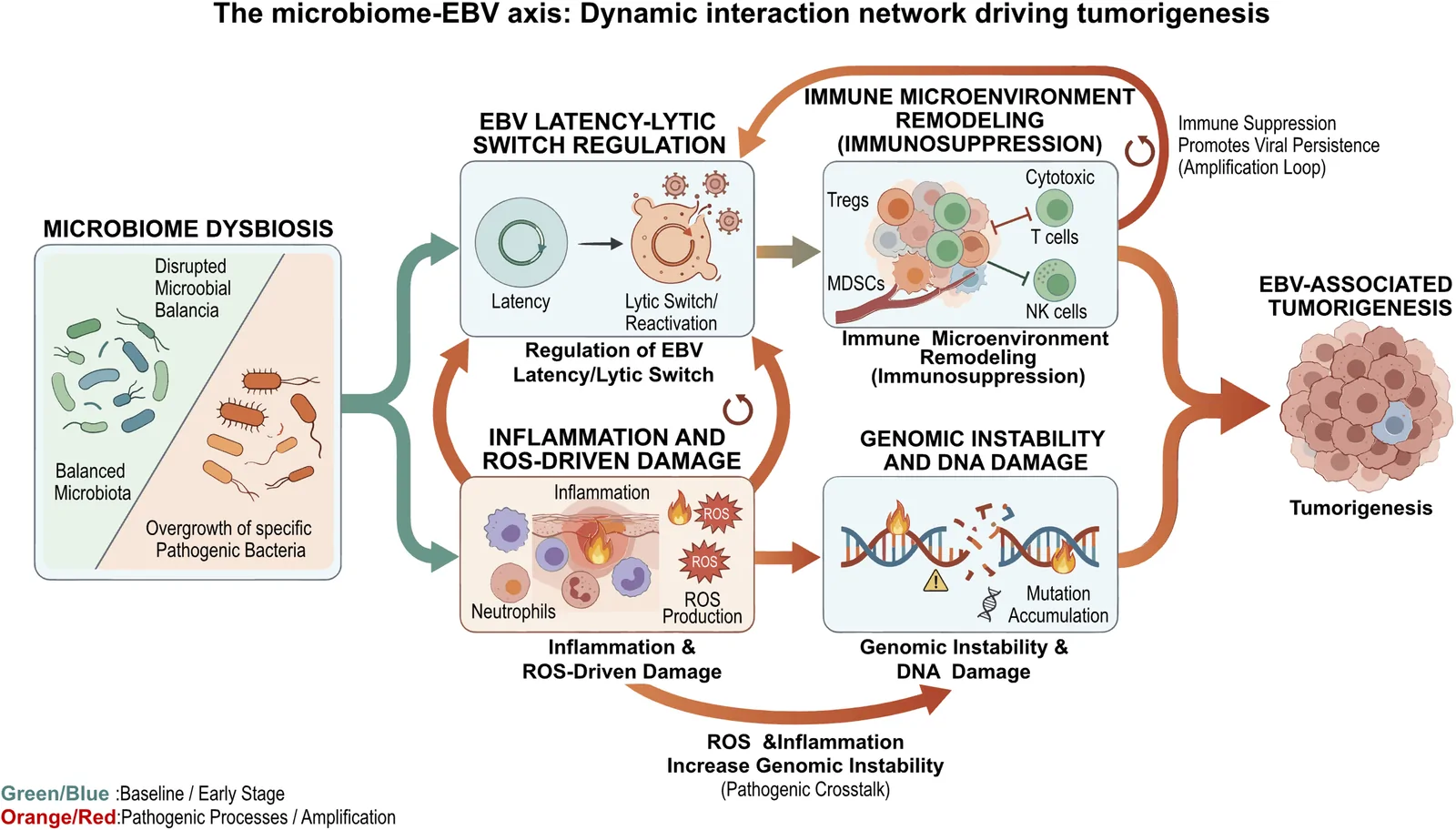
Integrated theoretical framework of the microbiome–EBV axis in tumorigenesis. Conceptual network illustrating microbiome dysbiosis as the initiating event that drives four interconnected pathogenic modules: regulation of the EBV latency–lytic switch, remodeling of the immunosuppressive microenvironment, inflammation- and ROS-associated damage, and genomic instability. These pathways are not isolated but linked by crosstalk and positive feedback, whereby inflammation promotes viral reactivation, immune suppression sustains viral persistence, and oxidative stress accelerates mutagenesis. Their coordinated amplification ultimately converges on EBV-associated tumorigenesis, providing a unifying systems-level framework for understanding how microbiome–virus–host interactions shape malignant transformation.

This review examines the potential role of the host microbiome in EBV tumorigenesis. These interactions are considered within the microbiome–EBV axis framework (**Figure 8**). Overall, the evidence reviewed here supports a model in which the microbiome may function as a context-dependent ecological regulator of EBV-associated tumorigenesis rather than merely a passive background variable. Across different levels of evidence, microbial communities and metabolites have been implicated in four interrelated processes: regulation of EBV latency and lytic reactivation, remodeling of the immunosuppressive microenvironment, inflammation- and ROS-associated damage, and genomic instability. However, the strength of support varies across these mechanisms. Some links are supported mainly by *in vitro* or animal models, others by observational clinical studies, and only a limited subset has reached the level of clinical validation.

This framework has both conceptual and translational implications. Conceptually, it extends the conventional virus–host model by incorporating microbial communities, microbial metabolites, and the tissue immune context as additional regulatory variables. Translationally, microbiome-derived signatures and metabolites may inform biomarker development and therapeutic strategies, although these applications remain incompletely validated.

Important limitations remain. Most clinical evidence is observational, and direct causal and interventional validation of the microbiome–EBV axis remains limited. Microbiome alterations may reflect tumor-associated ecological remodeling rather than initial drivers, and clinical intervention studies in EBV-associated cancers remain scarce. Future research integrating longitudinal clinical cohorts, multi-omics approaches, and rigorously evaluated microbiome-targeted interventions will be required to determine the clinical utility of this framework.
